# Conducting Interface for Efficient Growth of Vertically Aligned Carbon Nanotubes: Towards Nano-Engineered Carbon Composite

**DOI:** 10.3390/nano12132300

**Published:** 2022-07-04

**Authors:** Blagoj Karakashov, Martine Mayne-L’Hermite, Mathieu Pinault

**Affiliations:** NIMBE, CEA, CNRS, Université Paris-Saclay, 91191 Gif-sur-Yvette, France; blagoj.karakashov@cea.fr (B.K.); martine.mayne@cea.fr (M.M.-L.)

**Keywords:** vertically aligned carbon nanotubes, synthesis, chemical vapor deposition, physical vapor deposition, atomic layer deposition, conducting diffusion barrier layer, composite, carbon materials, composite properties

## Abstract

Vertically aligned carbon nanotubes (VACNT) are manufactured nanomaterials with excellent properties and great potential for numerous applications. Recently, research has intensified toward achieving VACNT synthesis on different planar and non-planar substrates of various natures, mainly dependent on the user-defined application. Indeed, VACNT growth has to be adjusted and optimized according to the substrate nature and shape to reach the requirements for the application envisaged. To date, different substrates have been decorated with VACNT, involving the use of diffusion barrier layers (DBLs) that are often insulating, such as SiO_2_ or Al_2_O_3_. These commonly used DBLs limit the conducting and other vital physico-chemical properties of the final nanomaterial composite. One interesting route to improve the contact resistance of VACNT on a substrate surface and the deficient composite properties is the development of semi-/conducting interlayers. The present review summarizes different methods and techniques for the deposition of suitable conducting interfaces and controlled growth of VACNT on diverse flat and 3-D fibrous substrates. Apart from exhibiting a catalytic efficiency, the DBL can generate a conducting and adhesive interface involving performance enhancements in VACNT composites. The abilities of different conducting interlayers are compared for VACNT growth and subsequent composite properties. A conducting interface is also emphasized for the synthesis of VACNT on carbonaceous substrates in order to produce cost-effective and high-performance nano-engineered carbon composites.

## 1. Introduction

### 1.1. Importance of Carbon Nanotubes (CNT) in the Development of Innovative Materials

The properties of its components govern the performance of any composite material. Different assemblies of CNT, seen as hollow cylindrical carbon structures of sp^2^ carbon, are indeed becoming part of the composite world within many industrial areas due to their remarkable properties. As previously reported [1,2,3], these manufactured carbon nanostructures are characterized and evaluated to exhibit extreme thermal and electrical conduction, mechanical performance, and many other extraordinary properties in various applications [4,5].

Once observed to possess highly interesting properties, today’s research focuses on the effective production of CNT with different properties and arrangements, thus, expanding their application portfolio. The effective production of CNT is of high importance to develop industrial-grade, ready-to-use, CNT materials and overpass many challenges concerning production cost, energy consumption, environmental sustainability, material concept, and performance [6,7,8,9]. One of many, the VACNT, also known as CNT forests, arrays, or carpets, are a dense network of CNT commonly aligned in a direction perpendicular to a substrate used for the growth [9,10].

### 1.2. Vertically Aligned Carbon Nanotubes (VACNT)—CNT Assembly with Specific Advanced Properties

Made of billions of vertically aligned CNT per centimeter square area, typically ~10^9^–10^12^ VACNT/cm^2^ [11], the VACNT show exclusive potential for improving the surface area, chemical and thermal stability, electrical conductivity, and other properties of different materials and their final use [8,12,13,14,15,16]. 

Mainly, VACNT are directly grown on suitable substrates using the chemical vapor deposition (CVD) technique [17,18,19,20,21,22,23]. The preference of this ahead of other known methods is due to multiple advantages, such as easily modified, scalable and straightforward way, performed in a wide range of pressure and temperature, and giving rise to cost-effective and high-quality VACNT [24,25]. Simultaneously, the same techniques can be used to modify and prepare the substrate to achieve the growth of VACNT and to tune the final properties of the composite material. In general, CVD growth of VACNT is performed through the catalytic decomposition of vaporized carbon precursor on a surface of a substrate, pre-decorated with catalyst nanoparticles, all performed at sufficient activation temperature, adjusted atmosphere, and pressure [3]. Well-known metal catalysts, such as Fe, Ni, Co, or alloys, are heat-treated at temperatures between 600 and 1100 °C in a reduction gas environment to facilitate the formation of catalyst nanoparticles [20]. Different gas, liquid, and solid carbon sources have been thermally evaporated or directly introduced for the growth of carbon nanotubes on present catalyst nanoparticles [21]. For example, C_2_H_2_, CH_4_, CO, C_2_H_4_, benzene, camphor, and ethanol are commonly used carbon sources. In parallel, many other parameters are well-detailed in the literature for the efficient CVD growth of VACNT [21].

When performing pure thermal CVD (unlike plasma-enhanced CVD or other), the sufficiently dense layer of catalyst nanoparticles initiates aligned growth of the CNT network, perpendicular to a flat substrate and owing to the crowding effect [26]. Though numerous CVD techniques present promising results for the growth of VACNT, aerosol-assisted catalytic chemical vapor deposition (AACCVD) is one of the most robust, easy-to-upscale [27], and straightforward CVD processes. Advantageously, a single-step AACCVD process simultaneously introduces the catalyst and the carbon precursor in the growth zone, allowing continuous restoration of poisoned catalyst and growth of VACNT [28,29,30,31], especially at relatively high temperatures (e.g., 800 °C and beyond).

Although the growth of VACNT via CVD is by far the most effective method, with multiple tuning options for substrate pretreatment, there are still some bottleneck steps to be tailored. Catalyst nanoparticle size/density and effectiveness are critical features for the appropriate growth of VACNT [29]. Part of many reasons for the distortion of the above-mentioned catalyst properties could be the subsurface diffusion within the support layer. The activity and lifespan of CNT catalysts can also be impacted via the Ostwald ripening effect (i.e., regrouping of nanosize catalyst particles giving rise to larger ones) due to the morphology/structure of the used template [8,32,33]. Thus, the former discoveries underline the importance of the choice of catalyst and substrate, with-/-out interface layers, for the growth of VACNT. 

### 1.3. Substrates for Growth of VACNT

Substrates of different nature and morphology are evidenced as suitable for the growth of VACNT due to easy-to-analyze or easy-to-industrialize properties. Various substrates have been used for the growth of VACNT, such as silicon wafers [9,24,31,34,35], quartz [36], aluminium foil [13,37,38], piezoelectric aluminium nitride film [39], stainless steel [40], titanium wire [41], magnesia and magnesium aluminate [42], as well as carbon fiber cloth [36,43,44,45] or paper [46,47], graphite [5,48], graphene paper [49] or oxide [50], reticulated carbon foam [51], and many others [21,52]. Among all, commercial fibrous carbon 3D substrates (CF tow, cloth, felt, paper, or other woven or non-woven fabrics) are advantageous since they possess exceptionally high mechanical strength and well-defined anisotropic thermal and electrical properties [53]. In addition, the fast development of 3D nano- to macro-scale composites by the growth of VACNT on fibrous carbon substrate presents even greater interest among the research and industry communities. Compared to various planar substrates, most fibrous carbons are flexible and porous matrixes with improved specific surface area, desired to prepare hierarchical composites in many applications. Respectively, the importance of various CF-CNT and CF-VACNT hybrids have been reported in scientific publications and patents [54,55]. 

However, not all substrates are suitable to support the required growth of VACNT. For example, Fe, Ni, or Co catalyst used for the direct growth of VACNT on Si is suppressed due to metal silicide or metal silicate formation at the synthesis temperature, i.e., the catalyst fails its primal activity [56]. Thus, the selection of the substrate nature mainly depends on the foreseen use and its compatibility to support the growth of VACNT with designed properties. Hence, some substrates/templates can only be used once covered with a suitable diffusion barrier layer (DBL), also known as interface, buffer, or even adhesion layer, interlayer or underlayer, and even catalyst support layer that promotes the growth of VACNT. Depending on its properties, one DBL should improve the catalyst activity (without alloying with the catalyst) and suppress its diffusion within the substrate at temperatures matching the nanoparticles formation and VACNT growth processes. Additionally, one DBL can promote adhesion and conductivity between the neighboring interfaces (substrate/DBL/catalyst/VACNT).

### 1.4. DBL towards Enhanced Growth of VACNT on Diverse Flat and 3-D Fibrous Substrates

Although beneficial for the homogeneous deposition of catalyst nanoparticles/growth of mm scale VACNT, most of the DBL used to date (such as Al_2_O_3_, SiO_2_, or MgO) [33,35,57,58] are generally insulators that limit the conducting contact between the VACNT and the substrate [59,60,61]. Unlike insulating DBL materials, semi-/-conducting DBLs show higher activity with the catalyst layer, such as interdiffusion and alloy formation that further affects the dewetting of the catalyst layer into a dense network of nanoparticles [62]. Therefore, different research groups are working on developing improved semi-/-conducting DBL for the growth of lengthy, dense, and tailored VACNT. Both semi-conducting and conducting DBL are suitable for final targeted applications compared to the generally insulating equivalents. Hence, in the following review, we will focus on both conducting and semiconducting material (hereunder mentioned as ‘conducting’) candidates for DBL. 

Lately, the growth of VACNT have been reported on different conducting DBL materials, such as aluminium [4,23,25,46,51,56,63,64,65,66,67,68,69,70,71,72,73], molybdenum aluminide [34,74], tantalum [62,75,76], tantalum nitride [77,78], titanium [79,80], titanium aluminide [47], titanium nitride [60,81,82,83,84,85,86,87,88], metal silicide nitride [89,90,91], their stacking use, graphitic layer [92] and many others. Nowadays, researchers are using physical vapor deposition (PVD), atomic layer deposition (ALD) [86,93,94], or CVD techniques for the deposition of various conducting DBL. Moreover, another interest of study is the influence of DBL morphological, structural, and chemical properties over the catalyst layer and the growth of VACNT [9]. An additional challenge is the growth of VACNT on 3-D and flexible substrates with the deposition of homogeneous DBL, especially for applications that mandate good electrical, thermal transport, and mechanical properties [48]. 

To the best of our knowledge, it is critical to obtain an up-to-date comprehensive review that highlights and summarizes the development and use of conducting DBL for VACNT growth. Within the increasing demand for the development and fast application of high-performing composites, this review details the available methods to achieve the growth of VACNT in conducting DBLs. Special attention is given to the growth of VACNT on carbonaceous substrates and the essential use of suitable conducting DBL. Therefore, the review highlights the importance of the initial materials selection, substrate preparation, DBL deposition, and CVD procedures under which the growth of VACNT is performed, all playing a critical role in producing effective composites. The presented review should simplify the way for controllable and further up-scalable production of multidimensional VACNT composites within various applications.

## 2. Conducting DBL Solution for the Growth of VACNT on Flat and 3-D Fibrous Substrates

In this section, a discussion is developed on the production, properties, and use of conducting DBLs (compared to SiO_2_ and Al_2_O_3_) shown to provide growth of VACNT of different morphology and properties.

### 2.1. Via Chemical Vapour Deposition (CVD)

As introduced, the CVD method is widely used to synthesize VACNT on predefined substrate/DBL systems with reasonable control of the growth parameters. Keeping this in mind, the use of CVD to deposit DBL with improved conductivity properties is one viable solution toward double–step single-system growth of VACNT. Well-defined deposition of SiO_x_ via flow injection/evaporation in CVD is already performed to deposit DBL on carbon fiber substrates for the growth of VACNT [36,43,44,95,96,97,98,99,100]. The process yields the deposition of ceramic DBL from organometallic precursors, also used on other substrates, such as stainless steel, palladium, ceramics, or any other substrates compatible with the temperature of VACNT growth [43,97]. Yet, the electronic interaction is restricted at the surface/interface and determined by the differences in electronegativity on insulating DBL, such as SiO_2_. On the contrary, long-range charge redistribution, driven by the dissimilarity of Fermi energies, is observed on other semi-/-conducting oxides (TiO_2_, Va_2_O_5_, RuO_2,_ and others) [101]. For example, integrating transition metal oxides on VACNT composites enhances the energy conversion and storage application [25]. Thus, today’s research is also focused on the use of different transition metal oxides, such as TiO_2_ [40], V_2_O_5_ [102,103], RuO_2_ [104], or MnO_2_ [105] for the development of different substrate/(VA)-CNT supercapacitors with improved discharge-charge profiles and attractive capacitance values. 

CVD and its effects on the deposition of metal oxide/nitride coatings on the different substrates have been intensely investigated [106,107]. However, the deposition of the mentioned or other semi-/-conducting oxides/nitride DBLs (via CVD) for the growth of VACNT on flat or 3-D substrates is a topic that demands further attention. Some of the possible reasons for the low dual use of the CVD system is the necessity to be equipped with a flow injection/evaporation system as in ACCCVD for the successive deposition of DBL and the introduction of carbon source/catalyst precursor for the VACNT growth. Thus, most of the cited studies outline the need to perform deposition/coating and annealing of a barrier layer and the deposition of catalyst top-layer prior to the transfer in a CVD chamber for the growth of VACNT. Bottleneck points can be the specified precursor/substrate properties and deposition conditions as the nature/stability of substrate—DBL precursor, the need for reactive/inert atmosphere, predefined deposition temperature, and post DBL deposition thermal treatments. Other properties can also be defined as crucial toward the growth of homogeneous film, with adequate structural and conductivity properties.

Moreover, the advantageous use of organometallic compound precursors is described as the perspective for developing CVD deposition of semi-/-conducting oxides/nitride thin films [103,106,108]. For example, transition metal nitrides, MNx (M = Ti, Zr, Hf, V, Ta, Cr, Mo, and W), deposited via CVD deposition with stoichiometry are refractory materials that show metallic conductivity, excellent chemical durability, and enhanced optical properties [109]. Moreover, research has been accomplished to improve the deposition of thin films with an improved crystalline structure, purity, and conductivity properties using metal-doping or decomposition reaction modification additives [109,110,111].

### 2.2. Via Physical Vapour Deposition (PVD)

Metal oxides and metals/metal alloys are evaporated or sputtered onto a substrate as a protective top-cover to prepare composite (stacking) materials. Thus, electron gun (e-beam) evaporation and magnetron sputtering are the most commonly used PVD methods. This technique generally forms pure coatings only on the plasma-exposed substrate face, with different and mainly superior structural properties when compared to the same via CVD or ALD. Therefore, PVD is widely considered a suitable technique for preparing DBL with appropriate morphology and structural properties, required as a catalyst supporting layers for the growth of VACNT on different substrates. However, performing a PVD deposition of a DBL demands an additional production step before the CVD growth of VACNT. Hence, it is possible to oxidize or introduce impurities on the DBL surface before the final use.

**Figure 1:**
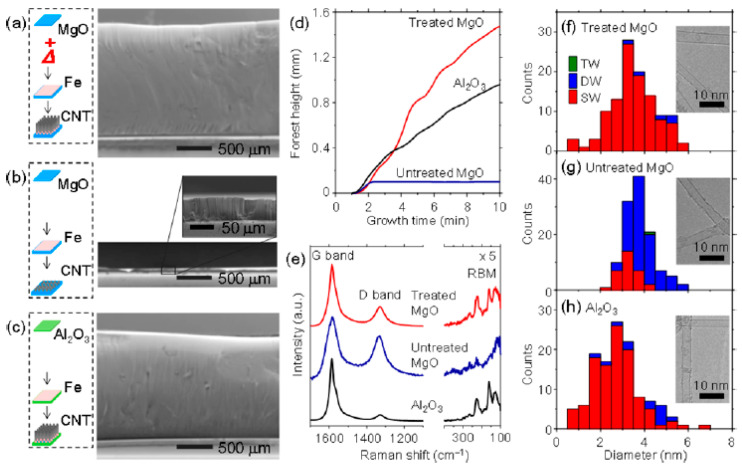
SEM micrographs of the VACNT grown on (**a**) annealed MgO, (**b**) MgO, and (**c**) Al_2_O_3_ DBL (left side inserts: scheme of preparation) with details of (**d**) CNT growth kinetics and CNT characterization via (**e**) Raman spectroscopy and (**f**–**h**) TEM. Reprinted with permission from Tsuji, Journal of the American Chemical Society; Ref. [35] copyright 2016, American Chemical Society.

As mentioned, few PVD metal oxides are well-known DBLs for the growth of VACNT. Al_2_0_3,_ SiO_2_, and recently, thermally post-annealed MgO are famous DBLs for the growth of dense, small-diameter single-wall (SW)-CNT carpets with a length of few hundred µm (Figure 1) [35]. Therefore, long VACNT are easily achievable on insulating DBLs, yet, the same needs to be developed for conducting ones.

Research teams have deeply investigated the use of Al DBL, with different post-deposition treatments, for the growth of VACNT [46,51,64,65,66,68,69,70,71,72]. 

**Figure 2:**
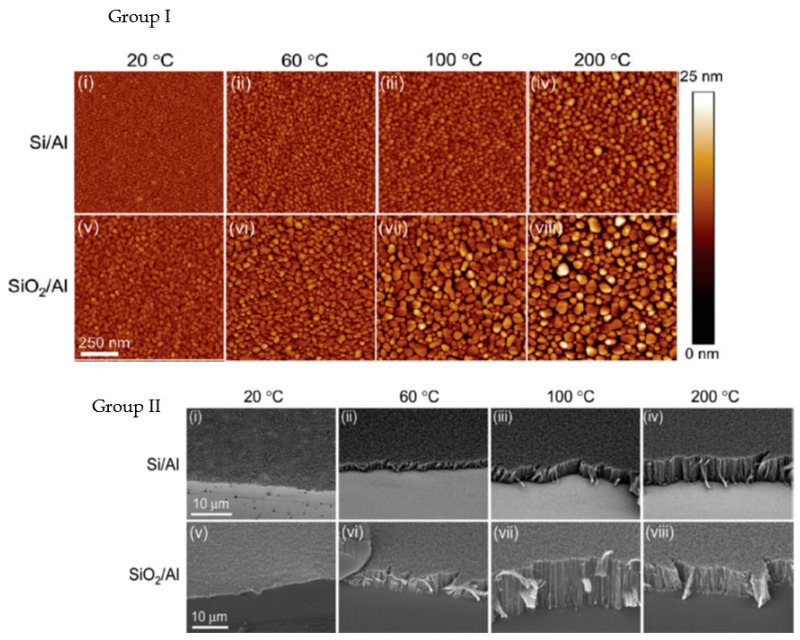
**Group I** (**i**–**viii**)—AFM tapping mode height micrographs of Si, i.e., native SiO_2_, (**i**–**iv**) and SiO_2_ (**v**–**viii**) with 10 nm Al DBL deposited at substrate temperatures of 20 (**i**,**v**), 60 (**ii**,**vi**), 100 (**iii**,**vii**), and 200 °C (**iv**,**viii**), all other conditions kept identical. **Group II** (**i**–**viii**)—SEM micrographs of single-wall VACNT growth on the substrates with DBL presented in Group I. Reprinted with permission from Burt, Journal of Physical Chemistry C; Ref. [65] copyright 2009, American Chemical Society.

As early as 2003, Ng et al. [64] presented Al DBL with Ni and Ni-Fe catalyst layer as an effective solution for the growth of short multi-walled VACNT. Burt et al. (2009) [65] investigated the deposition of Al DBL on the surface of native or introduced SiO_2_ as an effective way to grow single-wall VACNT with the use of Ni catalyst (detailed in Table 1). Modified morphologies of Al DBL were achieved using Si substrates with native or introduced SiO_2_ top-layer and the change of substrate temperature during the PVD process, other conditions kept identical (Figure 2, Group I). The CVD performance resulted in the growth of SWCNT, ranging from very short not-aligned to longer VACNT (Figure 2, Group II). The observed differences in the CNT properties were explained by the different DBL surface roughness and the level of Al oxidation (annealing process in the presence of oxide substrate) on the native SiO_2_ or the thick SiO_2_ (formed with performed wet thermal oxidation). No analyses were performed to determine the properties and the potential use of the resulting composites. 

**Figure 3:**
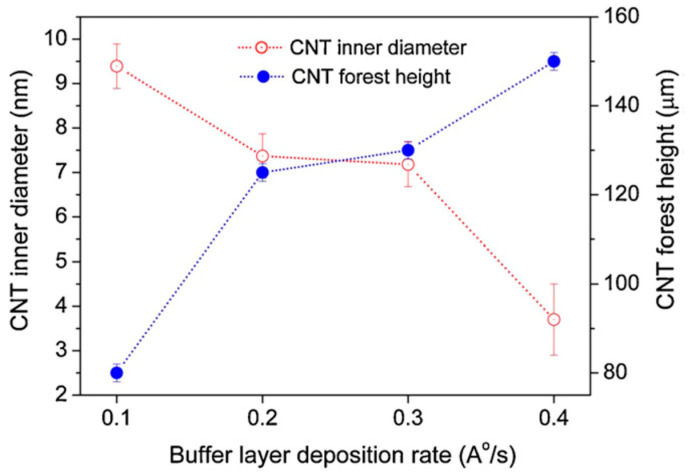
Al DBL deposited at defined rates over the inner diameter and height of grown VACNT. Reprinted with permission from Patole, Applied Surface Science; Ref. [68] copyright 2013, Elsevier.

Correlated to Burt et al. (2009) [65], Patole et al. (2013) [68] modified the grain size, i.e., the surface roughness of an Al DBL with the change of the deposition rate instead of the change of substrate temperature during PVD. Thus, the authors have revealed the DBL structure, morphology, and rearrangement (during the annealing and CNT synthesis process) to influence the growth of VACNT with modified CNT properties, such as CNT alignment, height, and diameter (details given in Table 1). Overall, the results present the beneficial use of large grains Al DBL topography (with enhanced rugosity) to support the formation of catalyst nanoparticles and allow higher carbon supply for the growth of small diameter, highly aligned, and long CNT. Even though not mentioned, the presence/formation of Al_2_O_3_ in the Al DBL could not be evaded due to the presence of native SiO_2_ and the post-PVD air transfer of the samples before the CVD process. The enhanced presence of Al_2_O_3_ is well defined by other authors [46,65,66]. For example, Choi et al. (2010) [66] considered the use of Al DBL for the formation of high-density Al_2_O_3_ and its effect on the formation of stable Fe_2_O_3_ catalytic nanoparticles for the growth of mm scale VACNT (Figure 3). The latter was achieved with the PVD of the DBL on a thick SiO_2_ surface and the use of CVD annealing (20 min)/CNT synthesis at a high temperature of 780 °C (details given in Table 1). The study generally presented a controllable way to optimize vertical alignment, growth rate, and density of VACNT by adjusting the DBL and catalyst layer thickness. The conductivity of the above-presented DBLs [65,66,68] should be heavily reduced compared to a pure Al film due to the enhanced oxidation of the Al for improved growth of VACNT. However, multiple studies confirm the necessity of a well-balanced compromise between the conductivity of a DBL (metal purity) and the vital presence of an oxide phase to anchor the active catalyst nanoparticles and endure the tunable growth of VACNT. 

**Figure 4:**
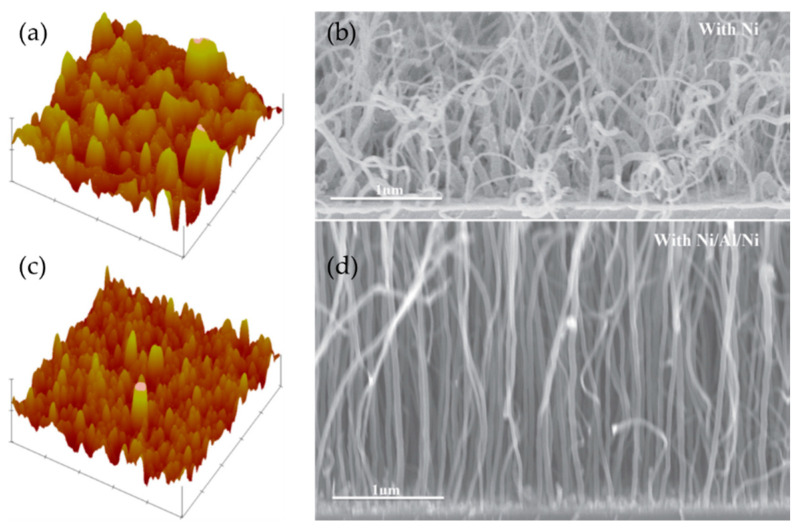
AFM representation of the top surface topography of (**a**) pure Ni and (**c**) multilayer catalyst/DBL/catalyst Ni/Al/Ni (scale bar 2 µm and 1µm, respectively). SEM micrographs of CNT grown on TiSi at 650 °C with (**b**) pure Ni catalyst and (**d**) multilayer Ni/Al/Ni interface. Reprinted with permission from Li, Diamond and Related Materials; Ref. [91] copyright 2017, Elsevier.

To advance conducting Al DBL in application composites, other teams have used different substrates and modified methods to decrease the unavoidable occurrence of native aluminium oxide [51,67,72,73,91]. To lower the contact resistance, Dijon et al. (2010) [67] have deposited Cu-Al (99.5%) alloy on Si wafer as DBL for the VACNT growth. The presence of Cu should have influenced the lower oxidation of the Al DBL surface. Additionally, the authors have de-oxidised (wet-etch method) the DBL before thin Fe catalyst film deposition. Thus, they observed a bottom contact resistance decrease of a factor of 100 when compared to the use of idem DBL without the de-oxidation step. However, they also concluded that the contact force of the AFM tip probe on the surface of the CNT carpet, i.e., change of contact area, has also influenced differences in the measured resistance (detailed in Table 2). Shah et al. (2013) [70] used low resistivity n-type silicon (100) wafers with a 300 nm thick Al DBL to grow short VACNT. Field emission studies present the improved current density of 20 mA cm^−2^ at a field of 3.5 V μm^−1^ with VACNT-Al DBL samples compared to 0.41 mA cm^−2^ of the directly grown VACNT sample. The reduction in contact resistance is attributed to Al DBL that formed thin Al_2_O_3_ film (offering an electron-tunneling effect) compared to the thicker native SiO_2_ layer on the lone Si substrate. Zhang et al. (2015) [51] performed the deposition of different thicknesses of Al DBL on Cu substrate to grow VACNT. Additionally, they investigated the interfacial adhesion properties of the complete composite (details given in Table 2). The authors performed floating catalyst CVD with a constant injection of active Fe nanoparticles on the DBL’s top-most surface. Moreover, thicker DBL was beneficial for the growth of the highest evidenced VACNT. The interfacial adhesion strength was observed to increase with the Al DBL thickness (from around 0.2 to 0.4 MPa for 10 to 30 nm Al DBL composites, respectively) using a lab-modified pull-off/compression test apparatus. However, the adhesion properties of the composite with Al DBL were much lower compared to the same with Al_2_O_3_ DBL. Thus, other studies (discussed later on) present the use of bi-metal (including Al) DBL to improve the overall composite mechanical properties. Zhong et al. (2016) [73] demonstrate the growth of high quality/density SWCNT forests on commercially available Cu foils via cold-wall CVD (details given in Table 2). The authors confirm the presence of thin native AlOx film on the Al DBL (due to air transfer and annealing process) that did not suppress the examined Ohmic contact between the SWCNT and the Cu substrate. The electrical properties of the SWCNT forests—Cu composite were investigated with a two-point probe station, yielding a very low overall resistivity of 60-80 Ohms. The electrical properties of the examined SWCNT forests were presented as highly promising for applications such as supercapacitors, batteries, and thermal interfaces. Pitkänen et al. (2015) [72] produced a library of conducting substrates with an Al interface effectively used for the growth of VACNT (details given in Table 2). A range of 5–10–20 nm was presented as effective DBL thickness for the growth of VACNT on all examined substrates. However, applying a 10 nm thick Al interface was found to support the longest VACNT on most of the studied substrates (Figure 1 within [72]). According to the electrical properties measurements, using an Al DBL (with the presence of thin native Al_2_O_3_ top-film) between the Fe catalyst and a conducting substrate did not increase the overall composite resistivity, compared to the same without the Al DBL. The latter indicates the possible use of the studied composites for the production of CNT-based electrodes, useful in many applications. For example, the total electrical resistance of VACNT-10nm Al DBL-Inconel^®^ 600 composite was found to be very low and below 10 Ω. Whereas, specific capacitances were measured to be 8.1 ± 0.6, 7.9 ± 0.4, and 10.0 ± 0.4 F g^−1^ for VACNT-10nm Al DBL on Inconel^®^ 600, Cu, and stainless steel substrate, respectively. Overall, the use of appropriate Al DBL on conducting substrate can be seen as a simple and tuneable solution to grow VACNT for various applications. Li et al. (2017) [91] proposed the use of multilayer Ni/Al/Ni catalyst/DBL/catalyst (sandwiched DBL) solution for the growth of VACNT via plasma-enhanced CVD (PECVD) on TiSi conducting substrate, at low temperature of 650 °C (details given in Table 2). The use of the Al interlayer improved the fine roughness of the top catalyst layer (prevent Ostwald ripening effect) and controlled the sub-surface diffusion of the catalyst in order to produce the growth of VACNT. On the other hand, the control sample with lone Ni catalyst did not form VACNT, all other conditions kept identical (Figure 4). The performed successive 2 nm Ni/1 nm Al/2 nm Ni PVD prevent the oxidation of the Al interlayer (up to the formation of Ni catalyst nanoparticles) that reinforced the Ohmic contact between the substrate and the VACNT, with a total resistance of 540 Ω. Moreover, the use of the presented or similar promising multilayers, with sandwiched Al DBL, was not tested with pure-thermal CVD to validate the growth of VACNT without the plasma-enhanced alignment effect on the CNT.

Other authors have explored alternative routes to directly use high-surface-energy (higher than the one of Al_2_O_3_), conducting metals or metal alloys/silicides/nitrides/silicide-nitrides to achieve controlled growth of VACNT. The use of conducting DBLs is evidence of difficulty stabilizing metallic nanoparticles and the tendency of the catalyst to unfavorably alloy with and/or diffuse within the DBL at the conditions for VACNT growth. If the growth of VACNT is achieved on conducting DBL, the same are examined as less aligned, shorter/thicker CNT that form lower density carpets than an Al_2_O_3_ DBL. Therefore, the ultimate solution is still to be explored for different conducting DBL-catalyst combinations that meet the presented drawbacks.

**Figure 5:**
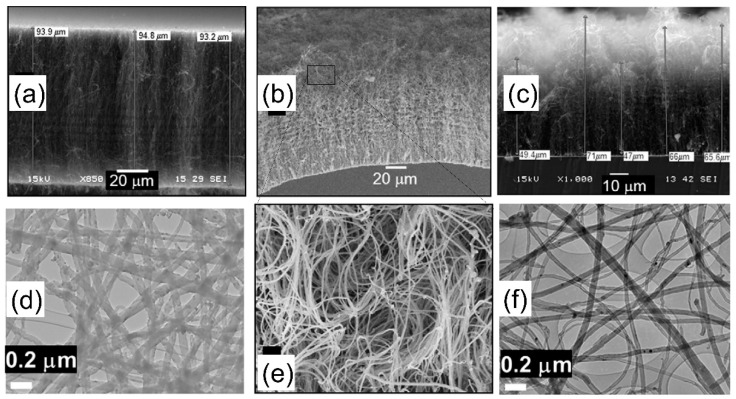
SEM micrographs of CNT carpets on (**a**) α-Ta(N) at 0.28 N/Ta at.%; (**b**) Ta_2_N at 1.2 N/Ta at.%; and (**c**) Ta_5_N_6_ at 1.65 N/Ta at.%. (**d**,**f**) are the TEM micrographs from CNT recovered from the sample presented in (**a**,**c**), respectively; (**e**) is the SEM magnification of a top part from the CNT carpet presented in (**b**) All CNT carpets were produced via AACCVD at 850 °C by using ferrocene in toluene as carbon precursor. Reproduced with permission from Bouchet-Fabre, Diamond and Related Materials; Ref. [77] copyright 2011, Elsevier.

**Figure 6:**
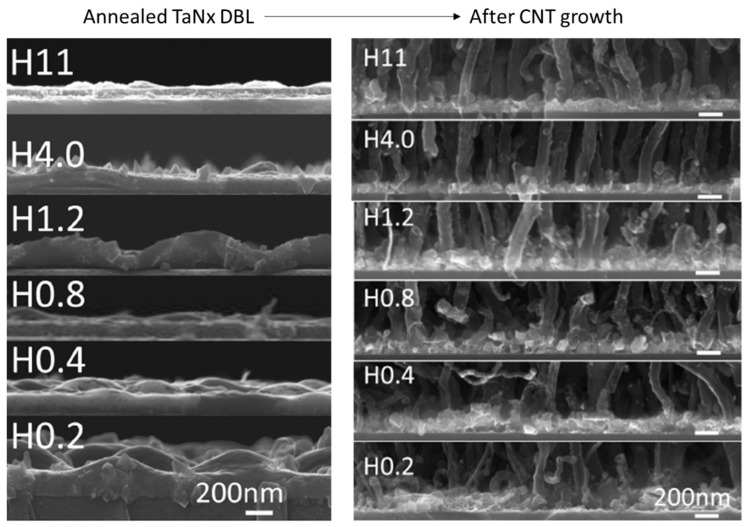
SEM micrographs (**left** column) for the variation range of TaNx DBLs after annealing under Ar flow and (**right** column) the same after successive growth of CNT with exposed DBL/CNT interface. Reproduced with permission from Bouchet-Fabre, Applied Surface Science; Ref. [78] copyright 2014, Elsevier.

PVD deposited Ta metallic layer was presented by Nessim et al. (2009) [62] (2010) [75] for the growth of 2 µm tall VACNT via fast-heat thermal CVD. The technique allowed preheating the reducing carrier gas and the carbon precursor in a hot zone before the growth zone to avoid thermal degradation of the used substrate and improve catalyst stabilization/activity, leading to successful VACNT growth (details found in Table 3). The same authors measured the electrical resistance of 2 µm-tall VACNT grown on Fe (5 nm)/Ta (30 nm) catalyst/DBL with the use of AFM, equipped with a metal-coated (Pt-Ir) conducting tip. The measured resistance, at around 35 kΩ, was defined as a good indication of good electrical contact between the CNT root and the metallic DBL. However, the previous study of Wang et al. [76] (and references within) has already shown a growth of VACNT of greater length (8–10 µm) on supports with Ta DBL via simple catalytic CVD. Moreover, the use of Ta as a DBL is observed as an insufficiently tunable method for enhanced growth of VACNT of high density and length. Thus, Ta as conducting DBL is explored through the use of TaN (Figure 5 and Figure 6). Our research group has already been working on developing TaNx DBL for the growth of VACNT via simple AACCVD on Si substrate. TaNx thin DBL have been deposited on Si via conventional [77] or high-power pulse [78] magnetron sputtering (details given in Table 3). The modification of the N_2_ flow during the PVD in reactive plasma N_2_/Ar with Ta target resulted in the deposition of DBLs with different N/Ta atomic ratios and structure that was observed to be altered after the annealing process [78]. The latter was evident to directly impact the catalyst settlement/nanoparticles formation/activity, i.e., the growth and properties of VACNT (Figure 6). Thus, neat and high-quality MWCNT forests were grown, with their diameter and length decreasing as the nitrogen content increased in the TaNx layers. Moreover, preformed material characterizations confirmed an exchange of the N atoms on the DBL surface with O atoms, found as residue in the CVD reactor. The presence of incorporated O atoms on the DBL surface proportionally increased with N atoms, yet always found to be 6 times lower. The oxidation of the TaNx, results in the formation of Ta_2_O_5_ in unstable under-stoichiometric α-Ta(N) phases, or a ternary segment of FeTa_2_O_6_ (during the CNT growth) at the stoichiometric nanocrystalline TaN phase, leading to CNT growth with different properties (shorter and larger or longer and thinner, respectively). Yet, the formation of oxide or ternary surface phases should significantly influence the electrical conductivity of the DBL and the overall composite. 

**Figure 7:**
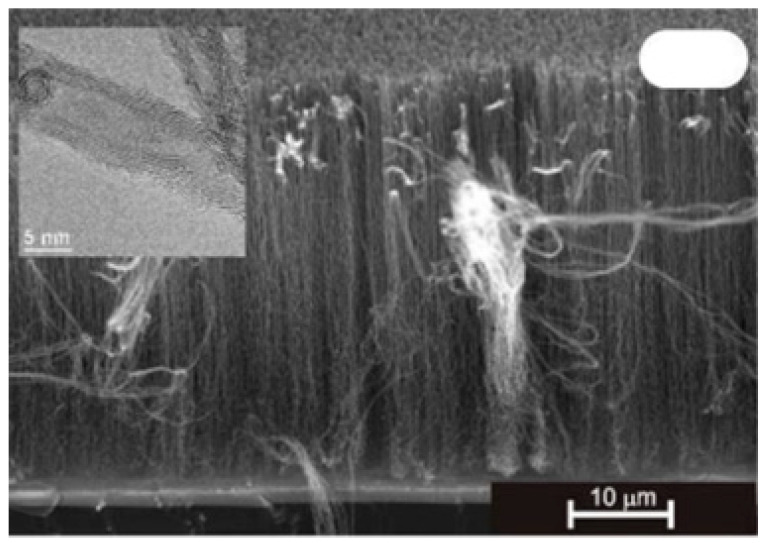
SEM micrograph of VACNT on 25 nm thick Co-Zr-N-(O) alloy DBL after CVD synthesis at 600 °C for 10 min; Reprinted with permission from Mierczynskia, Journal of Materials Science & Technology; Ref. [112] copyright 2018, Elsevier.

Mierczynskia et al. (2018) [112] have explored the influence of alloy composition, alloy concentration, and alloy thickness on the CNT growth and the impact of the CVD gas phase composition, temperature, and pressure. They detail the PVD of alloys with catalytic metal (Ct, such as Ni, Co, Fe, Pd) and transition metal (Me, such as Ti, V, Cr, Zr, Nb, Mo, Ta, W, Re) in an inert or a mixture of argon and nitrogen atmosphere for the achievement of conducting DBLs for the growth of VACNT. As previously published [113], the same team achieved a successful growth of well-developed VACNT only on Co-Zr-N-(O) alloy with specified properties of the DBL and CVD process of CNT growth (given in Table 3). Thus, they achieved growth of multi-layered VACNT with the rate of ~250 nm s^−1^ and a maximal CNT length of around 35 µm (Figure 7). Notably, the reduction in the DBL alloy during the annealing process was observed to decrease the presence and density of the top-surface active catalyst and suppress the growth of VACNT. The latter is connected with the reduced presence of native oxygen during the DBL restructuration at higher temperatures and in reactive NH_3_ atmosphere. The authors present the PVD deposition/CVD synthesis conditions with mutual importance for having both well-structured catalyst-DBL metal-alloy and successful VACNT growth process. Moreover, they confirm the complexity of the overall process due to the large number of factors that clearly have an immense impact on the CVD growth mechanism and demands further investigations towards full understanding.

**Figure 8:**
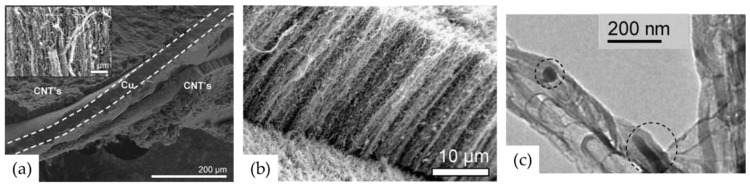
SEM micrographs of VACNT decorated on 20nm TiN DBL on Cu: (**a**) low with zoom insert and (**b**) high magnification of VACNT zone; (**c**) TEM images of recovered CNT samples with highlighted zones of Fe catalyst within the CNT inner core. Reprinted with permission from Garcia-Céspedes, Carbon; Ref. [83] copyright 2008, Elsevier.

Alternatively, titanium nitride, titanium silicide nitride, or aluminium silicide with attractive electronic properties are presented as conducting DBLs for the growth of VACNT for applications such as supercapacitors and micro-electronic devices/sensors/interconnectors. The use of conducting TiN DBL can be traced even as far as 2004. de los Arcos et al. (2004) [81] have grown VACNT by pure-thermal CVD using DBL such as Al_2_O_3_, TiN, and TiO_2_ with Fe catalyst. VACNT growth was presented only on Al_2_O_3_ and TiN with a growth rate of 5 and 2 µm min^−1^. Compared to the Al_2_O_3_ DBL, TiN did not support the growth of dense and well-aligned low-diameter CNT, yet it was recognized as promising to conduct DBL for further development. Subsequently, Garcia-Céspedes et al. (2009) [83] performed the deposition of TiN DBL on Cu substrate for the growth of VACNT via ferrocene injection (catalytic) CVD (details given in Table 4). The constant feed of active catalyst nanoparticles on the DBL surface resulted in areas of VACNT on the covered Cu faces, with a length between 20 and 30 μm and a dispersed diameter range between 10 and 100 nm (Figure 8). Hence, the thicker CNT were aligned, whereas the thinner ones were generally randomly orientated. The VACNT presented bamboo-like multi-wall morphology, with wall defects and a high presence of incorporated catalyst particles. Apart from the effect of the DBL, the low CNT carpet homogeny is presumably impacted by an asymmetrical dispersion of the Fe catalyst. However, the authors point out a null diffusion of the Fe catalyst into the TiN DBL by performing composition depth profiling via Auger electron spectroscopy and secondary ion mass spectrometry trace detection on the surface of the coated Cu substrates. Finally, the catalyst stability on the established TiN DBL roughness could have played an important role concerning the presented CNT properties. 

**Figure 9:**
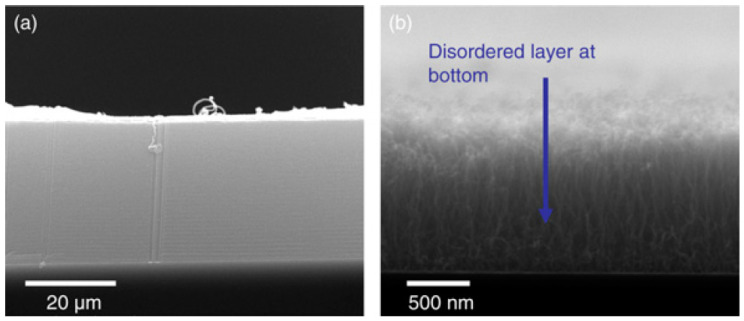
SEM micrographs of VACNT on (**a**) Al_2_O_3_ DBL/Fe catalyst or (**b**) TiN DBL/Fe catalyst. (**a**) highly dense and well-aligned compared to (**b**) highly disordered VACNT morphology. Reprinted with permission from Amama, Carbon; Ref. [85] copyright 2012, Elsevier.

**Figure 10:**
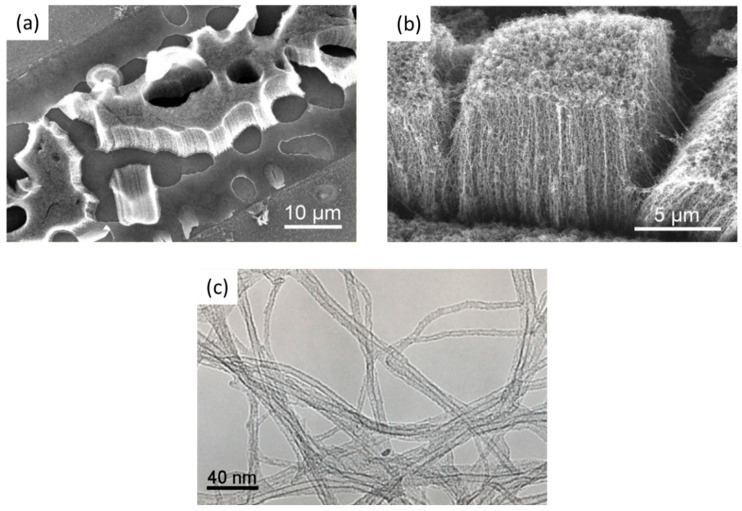
SEM micrographs of (**a**) the Si/TiN patterned substrate after the AC-CVD synthesis where (**b**) is the corresponding magnification of a growth zone of VACNT; (**c**) TEM image of SWCNT selectively grown on TiN surface. Modified after [88].

Amama et al. (2012) [85] reported TiN properties to promote catalyst stability/activity at a high number density and suppress subsurface diffusion to achieve VACNT growth on conducting DBL. The TiN DBL/Fe catalyst substrate composite supported VACNT growth, though, with much shorter and lower quality CNT due to the low activity and shorter catalyst lifetime than Al_2_O_3_ DBL (Figure 9). The observed CNT growth on TiN DBL was attributed to enhanced Ostwald ripening processes in the early growth phases and due to the observed subsurface diffusion of the catalyst. Moreover, the catalyst structural promotion during annealing and CNT growth processes is speculated to modify the interactions between the catalyst nanoparticles and the carbon precursor gas molecules. Finally, the authors do not exclude different results if other thermal CVD techniques are used instead of the cooled wall, hot filament CVD. In this context, Campo et al. (2019) [88] presented improved growth of denser and higher SWCNT forests via hot wall alcohol catalytic (AC-) CVD, compared to the previous results [85]. To increase the catalyst density and effectiveness, the authors used a dip-coating DBL deposition method of Mo-Co acetate precursor solutions (information on TiN DBL/catalyst deposition, CVD growth of VACNT, and the morphology of the latter are detailed in Table 4). Thus, the role of Mo is to suppress the coalescence of Co nanoparticles during the calcination process, the latter being the real catalyst for the growth of VACNT. Moreover, the growth of VACNT was not observed over some TiN DBL zones due to its low thickness (~200 nm) (Figure 10a) [88]. As observed previously [114], the authors evidenced VACNT absence when the TiN was neighboring a layer of SiO_2_, instead of sputtered Si, due to the higher surface free energy (Defined by the thermodynamics of surface states, in equilibrium, a solid composite tends to organize to the lowest surface free energy, thus, the catalyst favors a surface with a higher surface free energy, resulting in a stable composite system [88]) of SiO_2_ compared to TiN (64.7 and 40.6 mJ m^−2^, respectively). Therefore, both studies confirm that the properties of the used DBL and other surface layers in the vicinity (with uneven properties) can influence the preferential deposition/stabilization of the catalyst, i.e., the growth of VACNT. 

Yokoyama et al. (2007) [82] and Kpetsu et al. (2010) [84] achieved growth of VACNT with the use of Co and Ni catalysts on conducting TiN DBLs with 100 and 5 nm thicknesses, respectively, with the use of PECVD. However, PECVD restricts clear conclusions of the beneficial use of TiN DBL as effective solutions for the growth of VACNT.

A comparative study for pure TiN DBL deposited via PVD or ALD technique is detailed in Section 2.3, highlighting the advantageous use of ALD over PVD towards more effective catalyst stability and growth of VACNT [86].

**Figure 11:**
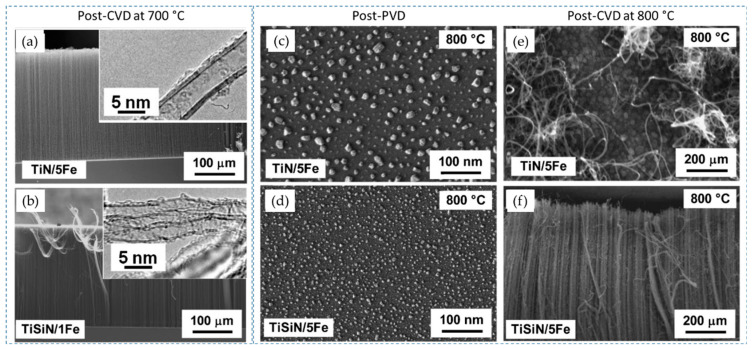
SEM micrographs of VACNT on (**a**) TiN with 5 nm Fe and (**b**) TiSiN with 1 nm Fe at 700 °C; Post-annealing catalyst restructuration on (**c**) TiN with 5 nm Fe film or (**d**) TiSiN with 5 nm Fe film at 800 °C, whereas (**e**,**f**) present the same after CNT growth at 800 °C, respectively; Reprinted with permission from Yang, physica status solidi (**b**) basic solid-state physics; Ref. [89] copyright 2014, John Wiley and Sons.

**Figure 12:**
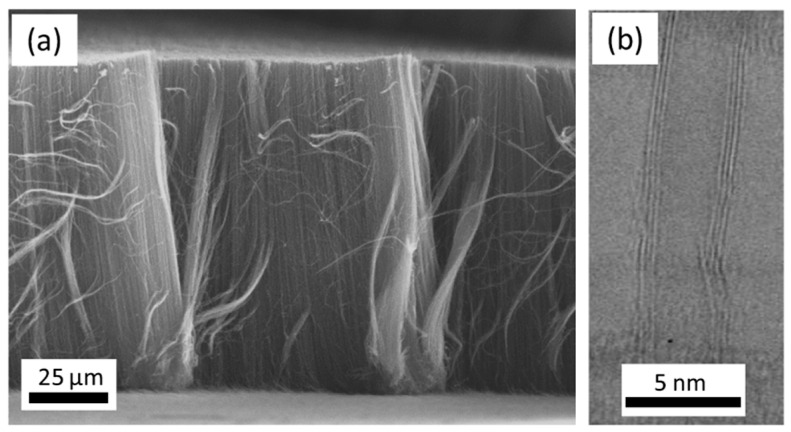
(**a**) Side-view SEM micrographs of VACNT achieved on TiSiN with 0.4 nm Fe/0.1 nm Al at 600 °C; (**b**) HRTEM image of recovered CNT sample; Reprinted with permission from Yang, Applied Physics Letters; Ref. [90] copyright 2015, AIP Publishing.

Differently, Yang et al. (2014) [89] have achieved robust growth of VACNT, with improved CNT density and length on TiSiN DBL, as compared to TiN of the identical thickness (Figure 11a,b). Due to a lack of grain boundaries, thin TiSiN films beneficially supported a prolonged catalytic activity of homogeneously-sized nanoparticles, kept immobilized throughout the CVD process. Consequently, the authors produced denser VACNT of low diameter CNT using 1 nm Fe catalyst film on TiSiN, compared to the thicker 5 nm catalyst film on TiN (details provided in Table 4). The TiSiN was also proven as an effective DBL to support Fe catalyst/VACNT even at a temperature of 800 °C (Figure 11d,f). Contrary, TiN DBL did not yield VACNT due to the enhanced Fe diffusion into the TiN bulk at the identical CVD conditions (Figure 11c,e). Moreover, TiSiN DBL substrate with low surface energy (less than 0.1 eV nm^−2^, compared to ~3.06 eV nm^−2^ for TiN [90]) disabled both subsurface diffusion and Ostwald ripening processes, allowing catalyst de-wetting into high-density nanoparticles (Figure 11c,d). The latter favored forest growth with area densities of the order ~10^12^ CNT cm^−2^, compared to ~10^10^ CNT cm^−2^ on TiN DBL, all other parameters kept identical. In both cases, Ohmic contact was examined between the substrate and the VACNT with resistance values of 0.8 and 3.4 kΩ for TiSiN and TiN, respectively. Yuan et al. (2015) [90] have also presented the effectiveness of matching TiSiN DBL with the use of ultra-thin Fe catalyst film and Al top-layer, with an analyzed thickness of 0.4 and 0.1 nm, respectively (details provided in Table 5). Furthermore, Al top-layer was evidenced to suppress the Fe catalyst surface (shallow) diffusion. Compared to their previous study results [89], the latter presented a superior areal density of thinner CNT with double/triple walls (Figure 12). Moreover, the irregular (post-annealing) ultrathin Al top-layer (with a native presence of alumina) was observed not to affect the composite resistance. Thus, the TiSiN is presented as an effective conducting DBL with matching or enhanced performance compared to some insulating DBL [115] as of CNT properties and areal density [89,90].

**Figure 13:**
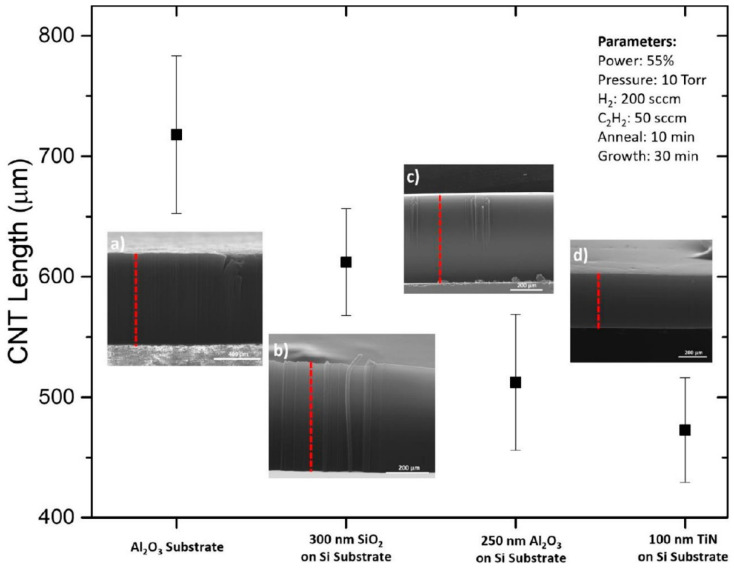
Length of VACNT (from 3 repetitions of each substrate) as an effect of the different DBL/thermal barrier layers. SEM micrograph inserts of (**a**) Al_2_O_3_ (**b**) 300 nm SiO_2_ on Si substrate (**c**) 250 nm Al_2_O_3_ on Si substrate (**d**) 100 nm TiN on Si substrate, where all four substrates had a 10 nm Al top-layer and a 3 nm Fe catalyst layer on the upper-most surface. Scale bar in (**a**) 400 µm, (**b**–**d**) 200 µm. Reported in [60].

**Figure 14:**
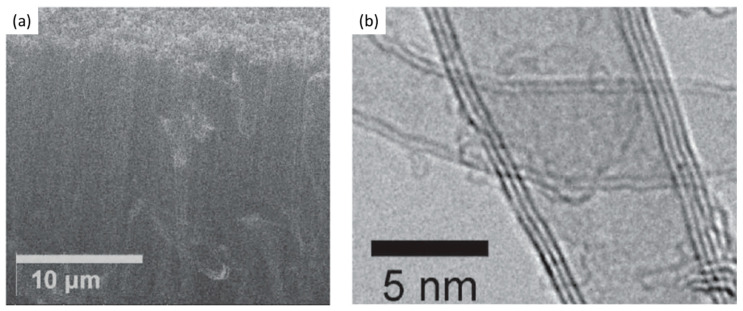
(**a**) SEM micrograph of VACNT grown on 100 nm TiN DBL at a substrate temperature of 400 °C; (**b**) TEM image of CNT taken from the identical sample. Modified after [87].

As presented by Yuan et al. (2015) [90], the potential use of different conducting DBL can be further improved by applied surface modifications, such as metal topping layer, that enhance the growth of VACNT with enhanced properties. Thus, well-defined VACNT have also been grown on bi-layer DBL, using Al top-layer. Moreover, researchers have investigated the use of Al DBL before introducing the catalyst or simultaneous sputtering of both catalyst and Al on metal/metal nitride DBL [34,60,67,71,74,87]. Thus, a thin capping layer of Al has been evidenced as a crucial part of one conducting DBL structure toward tunable growth of VACNT on different substrates. Multi-layer DBL is a beneficial solution to improve interlayer adhesion, suppress catalyst bulk diffusion and improve VACNT growth and properties, and at the same time retain good Ohmic contact between each composite component. Tas (2020) [60] and Ahmad et al. (2015) [87] introduced (additionally sputter-deposited) a thin Al top-layer (~10 nm) over the TiN before the annealing and VACNT growth processes. The Al topping layer was introduced to hinder the catalyst agglomeration during annealing, i.e., acting as an anchor for the formed catalyst nanoparticles. Moreover, the post-formation of Al_2_O_3_ from the Al top-layer is not excluded in both studies during the substrate annealing process. Though, the electric resistance of the presented combination is found to be as low as 10 Ω (as for as grown VACNT network probe), even after the annealing process and the possible formation of native Al_2_O_3_ [60]. Thus, TiN/Al solution is presented as an improved version of TiN alone with minimized interfacial resistance (TiN can be easily oxidized, adding resistance [79]) and improved catalyst stability/activity, reflected in tunable growth of high quality/length VACNT. A photo-thermal (PT)-CVD technique was used in both studies to grow VACNT. Figure 13 [60] and Figure 14 [87] compare and represent the length, and length and diameter, respectively, of VACNT grown on TiN DBL of 100 nm thickness. For [60], details of TiN DBL/Al top-layer/catalyst PVD deposition, CVD growth of VACNT, and the morphology of the latter are given in Table 5. The use of good electrically-conducting TiN as thermal insulation (8.92 W m^−1^ K^−1^ at 1000 °C) is highly suitable in PTCVD for protecting the substrate from severe thermal degradation. Thus, the substrates used in the cited studies are reported to stay at a very low temperature between 415–455 °C [60] and 350–440 °C [87] during the PTCVD growth of VACNT. Imperatively, TiN DBL and Al top-layer demonstrate good optical reflectance of infrared (IR) radiation (wavelength of 700–1000 nm) that further enhances the growth of VACNT on heated up, both from direct and reflected IR radiation.

Vollebregt et al. (2014) [71] used TiN DBL with Al/Co top-layer as a DBL top-cover and catalyst layer. The Co catalyst was beneficially used to grow VACNT at temperatures as low as 350 °C due to the need for much lower activation energy than for the Fe catalyst (details given in Table 5). Favorable, the presence of Al further enhanced the Co nanoparticle stability that resulted in VACNT. For example, the top layer of 3 nm alloy Co-Al (28%) supported VACNT of identical density compared to the 5 nm lone Co catalyst layer.

Bi-metal Mo/Al was presented as more promising in conducting DBL than other bi-metal solutions [34]. In addition, the use of unexplored Nb instead of Mo was observed as a possible alternative since both metals have similar oxidation resistance/mechanical properties at high temperatures and high melting points [116]. Mo alone did not present growth of CNT due to the strong alloying with the Fe catalyst and the loss of catalytic activity. The electrical resistance of the Mo DBL after the post-CNT growth process was examined to be lower than the same before the CVD process. In contrast, the use of a sole Al layer resulted in the growth of VACNT with high CNT areal density. Yet, the post-synthesis resistance of the Al layer was observed to be many orders of magnitude higher than the initial one. A possible explanation of the latter is the additional formation of a thick insulating oxide layer, hypothesized to be suppressed in the presence of Mo under-/-layer. The bi-metal Mo/Al DBL presented low contact resistance of 26 Ω (details in Table 5). The final VACNT/DBL/substrate composite exhibited electrochemical performances of 0.43 mF cm^−2^ at 50 mV s^−1^ with 92% retained cycling performance after 10 cycles. The supercapacitor composite also presented a power density of 0.28 W cm^−3^. Jiang et al. (2009) [34] also reported the method reproducibility with W/Al combination, i.e., generalizing the critical role of the Al top-layer for the enhanced growth of VACNT on conducting metal DBLs. Zang et al. (2018) [74] recently demonstrated the use of TiS_2_-covered VACNT, grown on 50/10/5 nm Mo/Al/Fe thin DBL, as an effective pseudocapacitive composite electrode with high capacitance and energy density (highest examined among comparable pseudocapacitors). 

Overall, the use of PVD Al DBL has been investigated through different studies showing the effect of the DBL surface morphology and structure, and the level of Al surface oxidation over the process of VACNT growth and their properties, or the properties of the final composite. In general, the vital presence of an oxide top-cover has been confirmed to anchor the active catalyst nanoparticles and enhance the growth of VACNT, regardless of its possible negative effect on the overall composite conductivity. Moreover, research teams have used different substrates and modified methods to decrease the unavoidable presence of native aluminium oxide to lower the presence of contact resistance.

Apart from the well-examined use of lone Al DBL, other authors have explored different conducting metals or metal alloys/silicides/nitrides/silicide-nitrides to achieve controlled VACNT growth. Finally, progress is made to use PVD conducting DBLs to boost conducting properties and simultaneously surface-stabilize catalytic nanoparticles with prolonged catalytic activity.

### 2.3. Via Atomic Layer Deposition (ALD)

Contrary to the frontal coverage with PVD, atomic layer deposition (ALD) could achieve dense and atomic-scale precision films on planar and non-planar substrates with screened surfaces. Recently, several studies have discussed the differences between an ALD and PVD (electron-beam or DC magnetron sputtering) techniques for the deposition of DBL and their later use for the growth of VACNT on flat and 3-D structures [86,93,117]. However, not many have presented the use of ALD for the deposition of DBL with improved conductivity properties. 

**Figure 15:**
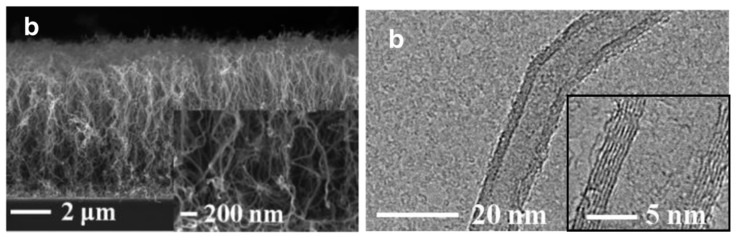
VACNT grown on TiO_2_ DBL at 650 °C, where: (**a**) presents side-view SEM micrograph of VACNT and (**b**) transition electron microscopy (TEM) of recovered CNT sample (with zoom insert). Modified after [93].

**Figure 16:**
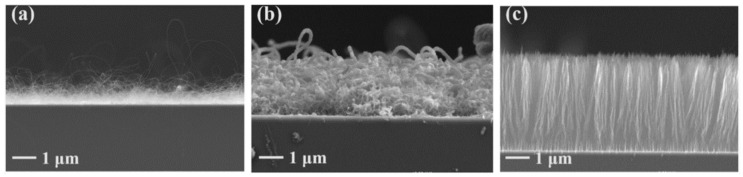
Cross-sectional SEM micrographs of CNT growth on the ZnO film at 800 °C, where: (**a**) presents the use of pure-thermal CVD in the presence of H_2_; (**b**) or (**c**) PECVD with H_2_ or NH_3_, respectively. Reprinted with permission from Yuan, Chemical Physics Letters; Ref. [94] copyright 2021, Elsevier.

**Figure 17:**
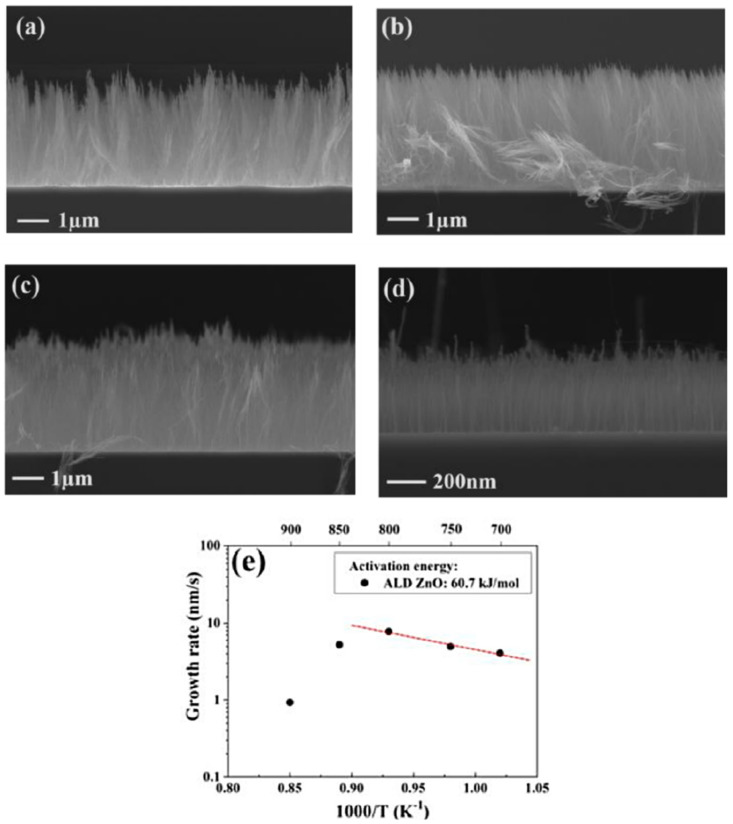
Cross-sectional SEM micrographs of CNT growth on the ZnO film at: (**a**) 700 °C; (**b**) 750 °C; (**c**) 850 °C; (**d**) 900 °C; and (**e**) presents the linear interpolation of the slopes from the growth rate of VACNT differences as a function of the deposition temperature, with an insert of the calculated activation energy. Reprinted with permission from Yuan, Chemical Physics Letters; Ref. [94] copyright 2021, Elsevier.

Contrary to the ineffective use of PVD TiO_2_ as DBL [81], Li et al. (2019) presented the use of ALD for the deposition of metal oxide TiO_2_ as DBL for the growth of VACNT [93]. Unlike the growth of long and dense VACNT carpets on Al_2_O_3_ and SiO_2_, the TiO_2_ DBL supported only short and low-density multi-walled CNT carpets with marginal alignment (Figure 15) due to severe sub-surface diffusion of the Fe catalyst nanoparticles or the high surface activation energy. Moreover, not all Fe nanoparticle catalysts achieved a CNT nucleation and growth due to the high surface activation energy on TiO_2_ DBL compared to SiO_2_ and Al_2_O_3_. The Ostwald ripening effect should have also influenced the density of the VACNT, yet to a lower order. The latter can be correlated with the presence of CNT of similar morphology on the tested DBLs. Multi-walled VACNT have been grown on ALD ZnO films only with PECVD [118] in the presence of NH_3_ [94], whereas the same was not achieved when pure-thermal and PECVD was performed in the presence of H_2_ atmosphere [93,94] (Figure 16). Yuan et al. (2021) [94] have presented the unsuccessful growth of VACNT with pure-thermal and PECVD in the H_2_ atmosphere only due to the high activation energy for the nucleation and initial growth of VACNT. The authors show the unmodified presence of Fe nanoparticles at different durations of the annealing process via morphology analysis of the catalyst-decorated DBL. The latter analysis and the CNT growth tests in different atmospheres strongly suggest the surface activation energy as the only barrier toward successful nucleation and initial growth of VACNT on ALD deposited and conducting ZnO films. The activation energy at 60.7 kJ mol^−1^ on the ALD ZnO film (Figure 17e) was found to be much higher when compared with the same on the ALD Al_2_O_3_ DBL, at about 39.1 kJ mol^−1^. Thus, the authors concluded the improved chemical reactivity of the carbon source (C_2_H_2_) in the presence of NH_2_ radicals than H radicals for the growth of CNT on Fe catalyst. Additionally, the VACNT growth is improved when synthesis temperature increases from 700 to 800 and is strongly reduced at 900 °C, related to enhanced Ostwald ripening and/or nanoparticles diffusion effects of the Fe catalyst (Figure 17a–d).

**Figure 18:**
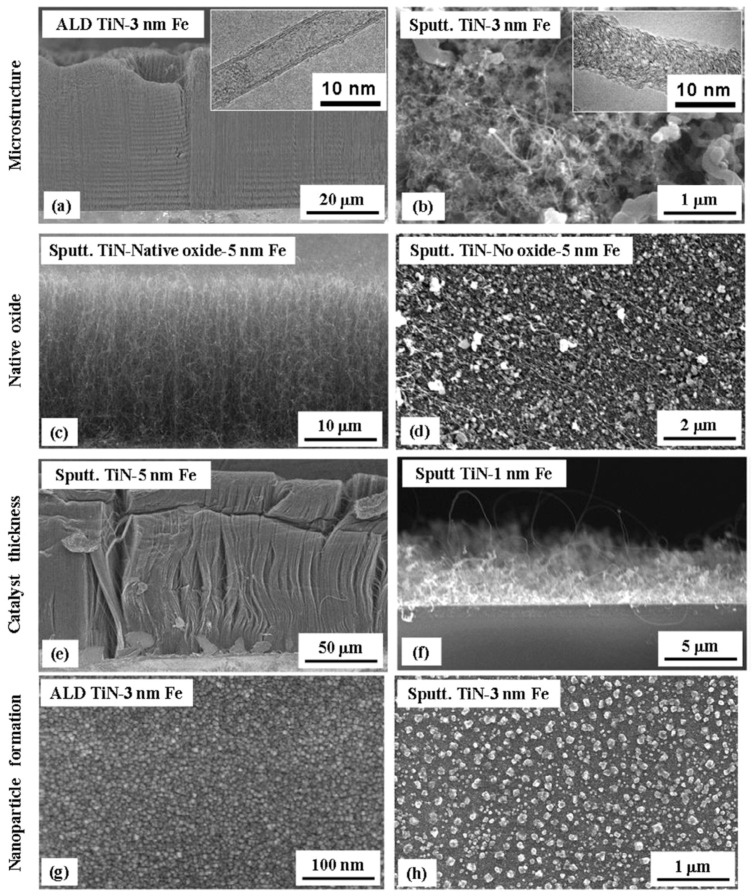
The effect of: (**a**,**b**) the microstructure (deposition technique); (**c**,**d**) presence of native oxide; catalyst thickness (**e**,**f**) over the growth of VACNT presented through a comparison of SEM micrographs. SEM images (**g**,**h**) present the density of Fe nanoparticles on DBL formed via the ALD or PVD process, respectively. Idem after growth of VACNT in (**a**,**b**). Note that the DBLs, shown (**c**) or (**e**), were PVD deposited at 10^−4^ or 10^−1^ mbar and provided different support of VACNT, respectively. Reprinted with permission from S.Esconjauregui, Carbon; Ref. [86] copyright 2014, Elsevier.

As for the use of transition metal nitride DBLs, TiN is a candidate of high interest with a low resistivity (~20 µΩcm) that should support ohmic contacts with grown CNT. Esconjauregui et al. (2014) studied the ALD deposition and use of TiN thin film with-/-out a native oxide layer and different thicknesses of the catalyst top-layer for the growth of VACNT via pure-thermal CVD [86]. Additionally, the authors compare the effect of ALD and PVD deposition on the film structure/morphology and its ability to support VACNT growth. The authors show that the growth of VACNT on TiN DBL depends simultaneously on the DBLs initial/post-annealing structure/morphology (as of deposition technique), the presence of surface native oxide (the surface free energy), and the Fe catalyst thickness (studied in the range from 0.5 to 5.0 nm). Differences have been observed in the nanoparticle size and density distributions, showing the dense presence of smaller size Fe nanoparticles on the ALD compared to the PVD deposited DBL (Figure 18g–h). The latter is attributed to the difference in the film crystallinity, i.e., highly crystalline TiN tends to restructure (cruck and surface rearrange) during a CVD process. Thus, a higher TiN crystallinity generates faster deactivation/diffusion of the Fe catalyst nuclei. ALD TiN film with a low-crystalline structure tends to preserve its microstructure even after the annealing process, which is beneficial for supporting dense Fe nanoparticle decoration (Figure 18a). The same was not evidenced with the highly crystalline PVD TiN (with an identical thickness of Fe top-layer as on ALD TiN, Figure 18b,f). PVD TiN needed thicker Fe film (as of 5 nm) to retain a sufficient number of Fe nanoparticles for the growth of VACNT (Figure 18c,e). In addition, the differences in PVD chamber pressure have been shown to influence the film microstructure, with more efficient DBL produced at a higher pressure of 10^−1^ mbar (Figure 18c,e). Another crucial parameter in DBL quality is the presence of a thin top layer of native oxide before the evaporation of the Fe catalyst layer (Figure 18c,d). The top-surface presence of TiO_2_ is attributed to the air exposure during the transfer process, analyzed/quantified via surface chemistry analysis. Lower-surface-energy TiO_2_ promoted the surface interactions while suppressing the bulk diffusion/interactions of the catalyst with the DBL. For densely-packed VACNT, an equilibrium balance should be found between the growth of VACNT and enhanced bulk diffusion/interactions (the native oxide is reduced and/or desorbed), both increasing at temperatures above 500 °C.

Details of the DBL deposition and VACNT synthesis/properties are summarized in Table 6 for all the above-mentioned studies using the ALD technique.

Finally, the use of ALD for the deposition of conducting DBL is an exciting approach to the growth of VACNT. Compared to other techniques, the presented advantages of ALD are the ability to perform conformal deposition of thin films on both planar and non-planar substrates with high precision and beneficial microstructure for the growth of VACNT. The ALD is still considered a highly time-consuming technique, even for the deposition of nanometric scale thin films. However, research is ongoing to develop alternative spatial ALD and its adjustment on continuous (roll to roll) manufacturing lines. 

## 3. Growth of VACNT on Carbon Substrates, with a Focus on CF Substrates

In this subsection, the review summarizes different approaches reported for the use of un-/modified carbon substrate or the same with predeposited DBL (via CVD or PVD) for the growth of VACNT. Even though few studies have examined the growth of VACNT on a carbonaceous substrate without the use of interface [5,32,48,49], promoted growth of tunable VACNT on CF is today achievable through the use of suitable DBL [4,36,43,44,47,95,96,97,98,99,100]. The cited and other references therein show that covering a CF substrate with an intermediate DBL can radically improve the growth of VACNT and prevent the possible degradation of the fiber structural properties. 

The absence of an up-to-date review of CF-VACNT materials, as for CF-carbon nanotube (CNT) composites [54], inspired us to dedicate a special section of this review to highlight the development of CF composites through the growth of VACNT.

### 3.1. Growth of VACNT on Carbon Substrates with-/-Out Surface Modifications

The ideal solution to obtain a VACNT without contact resistance and well adhered to the surface of a carbon substrate (by establishing carbon-carbon covalent bonds) is to directly deposit the catalyst nanoparticles without additional interlayers [5]. Unfortunately, the carbon substrate must own specific/defined structural properties to support the growth of VACNT. The structural properties of chemically stable carbon substrate are essential to suppress severe carbon-metal reactions (as metal interdiffusion) and suppress Ostwald ripening processes (retain well-dispersed nanocatalyst top layer) [5,32]. To the best of our knowledge, only a few studies examine the growth of the VACNT on commercial CF materials with specified structural properties. Despite today limited growth of the VACNT on uncovered commercial CF, the predeposition of a DBL is still observed as a critical synthesis point to achieve tunable growth of VACNT with remarkable properties.

The low number of studies concerning the direct growth of VACNT on carbon is directly related to the difficulty of controlling the homogeneous dispersion of catalyst nanoparticles on the surface of carbonaceous substrates [32]. The major drawback of the catalytic growth of VACNT on carbon is the interaction between a carbon substrate and catalytic nanoparticles, resulting in the subsurface diffusion and/or poisoning of the latter. On the other hand, the following studies encourage future research on the potential growth of VACNT on CF, the latter with improved graphitic structure. 

**Figure 19:**
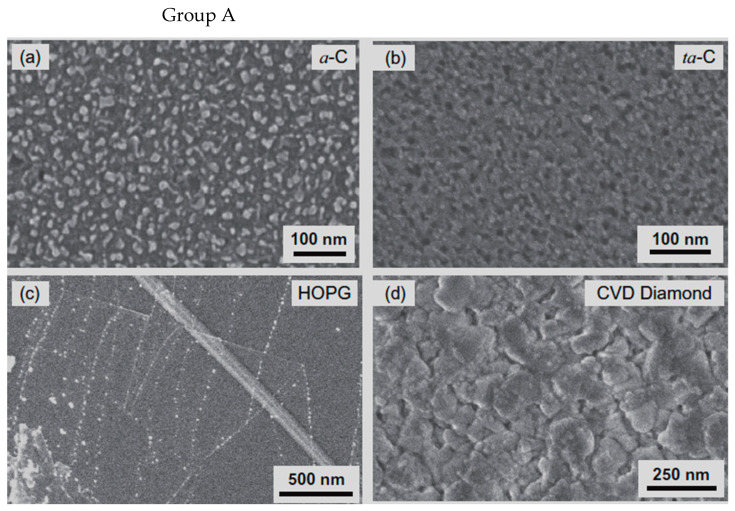

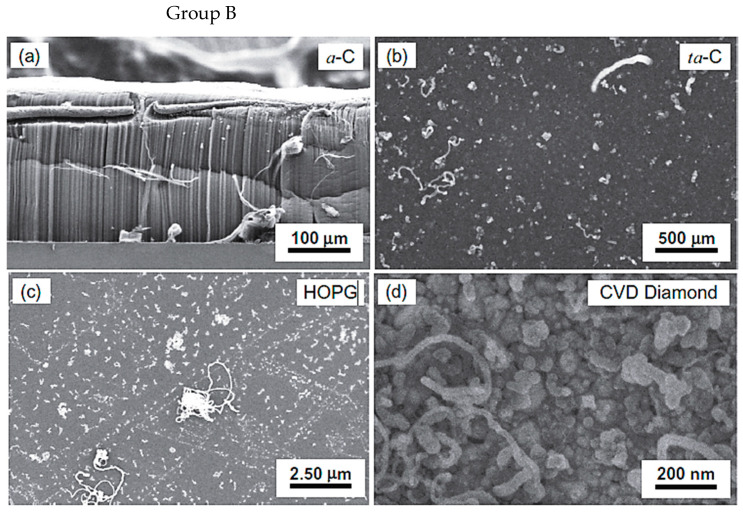
**Group A**—Top-view micrographs obtained by scanning electron microscopy (SEM) of Fe catalyst nanoparticles deposited on four different sp^2^:sp^3^ carbons: (**a**) (a-C, 85:15 sp^2^:sp^3^), (**b**) (ta-C, 30:70 sp^2^:sp^3^), (**c**) (HOPG, 100:0 sp^2^:sp^3^), and (d) (CVD diamond, 0:100 sp^2^:sp^3^). **Group B**—idem as in Group A after CVD synthesis of VACNT. Micrographs in **Group B**—(**a**) presents cross-section view, whereas (**b**–**d**) present top-view. Reprinted with permission from Cartwright, Carbon; Ref. [32] copyright 2014, Elsevier.

Cartwright et al. (2014) [32] showed that it is possible to retain a well-dispersed deposit of catalyst nanoparticles on the surface of a carbon substrate of defined sp^2^:sp^3^ ratio content (Figure 19 Group A). They also present the growth of VACNT of around 200 µm (Figure 19 Group B) via the base growth mechanism, indicating the strong adhesion of the Fe catalyst on the carbon substrate. Moreover, the authors present the boundaries for the direct growth of VACNT on different carbon substrates by varying the sp^2^:sp^3^ hybridization ratio. Thus, the authors performed CVD synthesis of VACNT on amorphous carbon (a-C, 85:15 sp^2^:sp^3^), tetrahedral amorphous carbon (ta-C, 30:70 sp^2^:sp^3^), highly oriented pyrolytic graphite (HOPG, 100:0 sp^2^:sp^3^), and CVD diamond substrate (0:100 sp^2^:sp^3^) (Figure 19 Group B). The results show the successful formation of catalyst nanoparticles and growth of VACNT on the ratio of 85:15 sp^2^:sp^3^ (a-C). After annealing at 750 °C in Ar and at atmospheric pressure, the predeposited Fe nanolayer on the a-C restructures into a highly populated zone (~3.2 × 10^10^) of nanoparticles, ranging from 10 to 22 nm (details for the substrate, catalyst deposition and CVD growth of VACNT are given in Table 7). On the contrary, the process yields low density, large Fe catalyst nanoparticles on the HOPG substrate, idem for the other substrates with the increased (or complete) sp^3^ carbon structure [32]. As for the HOPG, homogeneous dispersion of Fe nanoparticles is not observed only due to its completely flat surface, with only a few edge irregularities of the stacked graphitic planes [5]. Contrarily, the developed surface roughness of the ta-C and CVD diamond substrates indicate the enhanced diffusion of the Fe catalyst during the annealing process, suppressing the growth of dense, thin, long, and aligned CNT. The detailed morphological post-annealing changes are also correlated with the facility of excited Fe catalyst (or other catalysts) to decompose a single σ bond (with energy from 284–368 kJ mol^−1^) in sp^3^ carbon when compared to the double σ and π bond in sp^2^ carbon (with energy around 615 kJ mol^−1^). Thus, a substrate rich in sp^2^ carbon and, to some extent, irregular surface with adequate active sites possess a sufficiently inert surface and well-defined morphology to form a homogenous network of active Fe catalyst nanoparticles and further growth of VACNT. The same authors [32] highlight the importance of the catalyst pretreatment process, demonstrating that a reducing atmosphere (using H_2_ or NH_3_) can cause a catalytic carbon hydrogenation process and diffusion of the Fe nanoparticles beneath substrate surface. 

Yoneda et al. (2019) [5] performed a slightly different approach yet again emphasizing the importance of the rich graphitic structure and substrate surface roughness for the direct growth of VACNT on carbon materials (substrate preparation and growth/properties of VACNT can be found in Table 7). Instead of using pure HOPG with a flat surface (examined in [32]), they performed oxidative plasma treatment to produce densely protruded surface (Figure 1 within [5]), graphitic defects, and surface functional groups. The same authors successfully developed VACNT through the deposition of a dense network of Co or Fe catalysts on the modified HOPG substrate (Figure 4 within [5]). Moreover, the modified substrate morphology/chemistry suppresses the catalyst nanoparticle agglomeration and influences the formation of carbon-metal bonds (correlated with the presence of C=O functional groups) [5]. No diffusion of the catalyst nanoparticles was observed since the surface modification of the HOPG substrate did not modify the sp^2^ carbon nature of the substrate sub-surface. The final VACNT/CF composite presented specific capacitance observed to be higher than the same of the examined pristine HOPG and therein referenced composites (VACNT on TiN or Al DBL). Compared to referenced VACNT composites, the higher performance was observed due to much thinner CNT (10 nm of CNT thickness compared to 20 nm on TiN DBL), higher CNT density, and enhanced electrochemical active surface area. No information was given about the cycling performance and stability of the examined composite.

**Figure 20:**
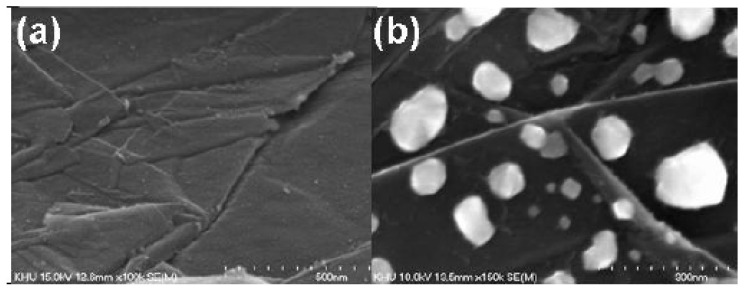
SEM images of (**a**) bare graphite foil substrate and (**b**) after Ni film deposition and agglomeration of catalyst nanoparticles during the annealing process, reported from [48].

**Figure 21:**
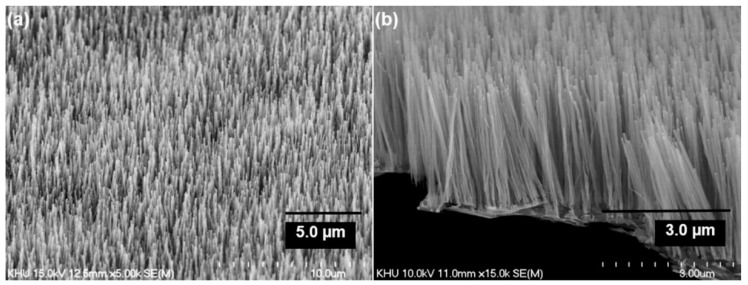
(**a**) Top- and (**b**) side-view SEM micrographs VACNT grown on flexible graphite foil via PECVD, reported from [48].

Even though performed via PECVD, i.e., the CVD process assists in the directional growth of CNT due to the attraction of the catalyst nanoparticles under plasma electrostatic forces [118], some research groups have successfully developed VACNT on graphite foil [48] or activated reduced graphene oxide (a-rGO) paper [50]. Ryu et al. (2014) [48] reported growth of VACNT on conducting and flexible commercial graphite foil, to be used as an all-carbon electrode under mechanical stress (conditions of substrate preparation and PECVD conditions for the growth of VACNT are provided in Table 7). The graphite foil surface was not modified before the PVD deposition of the Ni catalyst layer that, after annealing, resulted in the formation of nanoparticles. Different from the extremely flat surface of HOPG, the graphite foil composed of irregularly stacked graphene layers allows the homogeneous deposition of catalyst nanoparticles (Figure 20). Overall, the study presents a possible way to directly grow VACNT on a carbon substrate with the use of Ni catalyst (Figure 21). When compared to the use of graphitic foil alone, the all-carbon electrode (VACNT grown directly on a flexible graphite foil) demonstrated a higher electrochemical active surface area and higher electrocatalytic activity. In addition, the same authors performed flexibility tests to show that the examined all-carbon electrodes were bendable and non-breakable. The achieved strong and direct covalent bond of the CNT with the graphite substrate is defined to support the structural integrity during mechanical bending.

**Figure 22:**
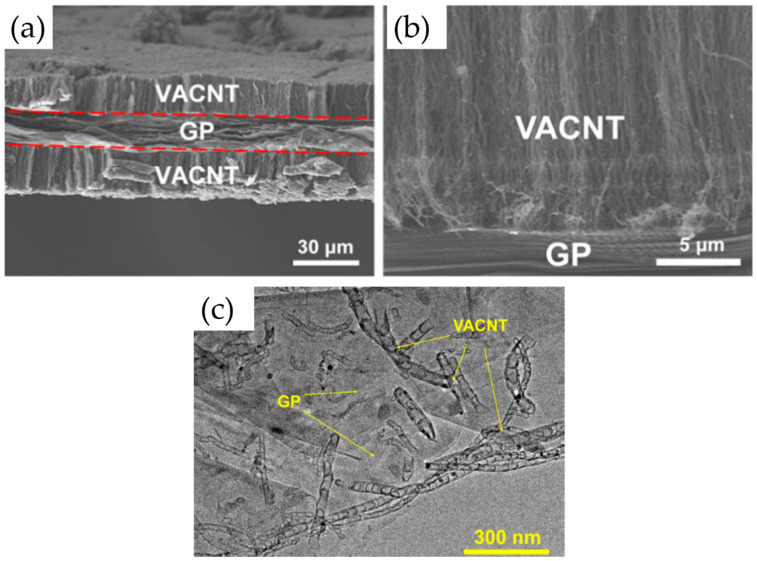
SEM images of (**a**) single layer of GP with VACNT and (**b**) magnification of the composite interphase. (**c**) TEM image GP sheets with grafted bamboo-like CNT following an ultrasonic sample preparation treatment. Adapted with permission from He, ACS Applied Nano Materials; Ref. [49] copyright 2022, American Chemical Society.

Very recently, He et al. (2022) [49] have presented high-performance advanced EMI shielding and thermal management multifunctional multilayer composite based on the VACNT-graphene paper (GP)/polydimethylsilane (PDMS). The authors detail the application performance of the cited composite due to its mechanical and conductivity properties and multi-layer structure (details given in Table 7). Whereas little is given for the CVD growth process of VACNT onto spruce GP and the structural characteristics of the GP substrate or the achieved VACNT (summarized in Table 7). GP is generally synthesized from a graphene oxide film, derived via the Hummer method, and additional film preparation processes [119]. The final result is a GP with a dense graphene flakes structure, closely stacked along the cross-section direction, presenting an essential wrinkle-like surface morphology. A well-prepared GP always presents a low defect level and good crystalline structure with crystallite size in the range of µm-scale and stacking order of graphene flakes similar to that of HOPG. As therein presented [49], the structural properties of the used GP have been sufficient to support the deposition of a dense network of Fe catalyst nanoparticles and the simultaneous growth of VACNT. Figure 22 presents SEM images of 20 μm height VACNT grown on two sides of a single layer of GP. Additionally, the TEM image (Figure 22c) details the solid interfacial adhesion of the bamboo-like CNT and GP sheets following an ultrasonic treatment. Once more, this study and the ones presented in this subsection clearly show the importance of an sp^2^-rich carbon substrate in combination with the irregular surface morphology, along with other methods and material parameters, to achieve VACNT without the use of a DBL.

**Figure 23:**
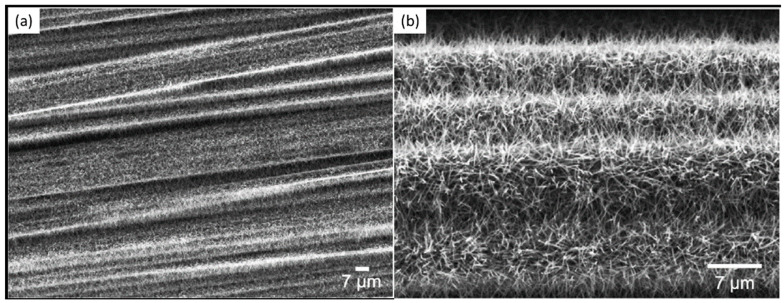
(**a**) CVD grown radially aligned CNT growths across CF yarn; (**b**) idem at higher magnification. Reprinted with permission from Li, Composites Science and Technology; Ref. [122] copyright 2015, Elsevier.

**Figure 24:**
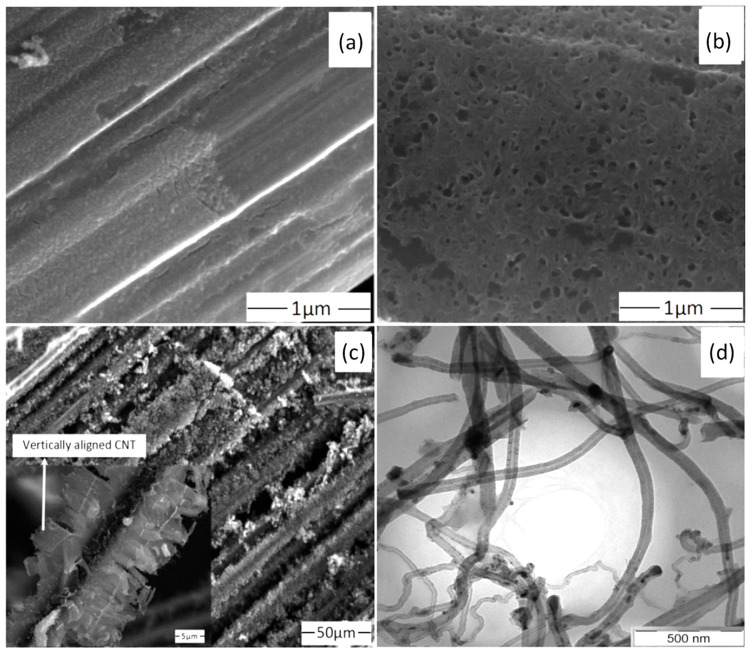
SEM micrographs of (**a**) before and (**b**) after calcination of CF coated with catalytic film, whereas (**c**) presents the same after CVD growth of VACNT with an insert at higher magnification. (**d**) TEM micrograph of CNT. Reprinted with permission from Rahmanian, Applied Surface Science; Ref. [120] copyright 2013, Elsevier.

**Figure 25:**
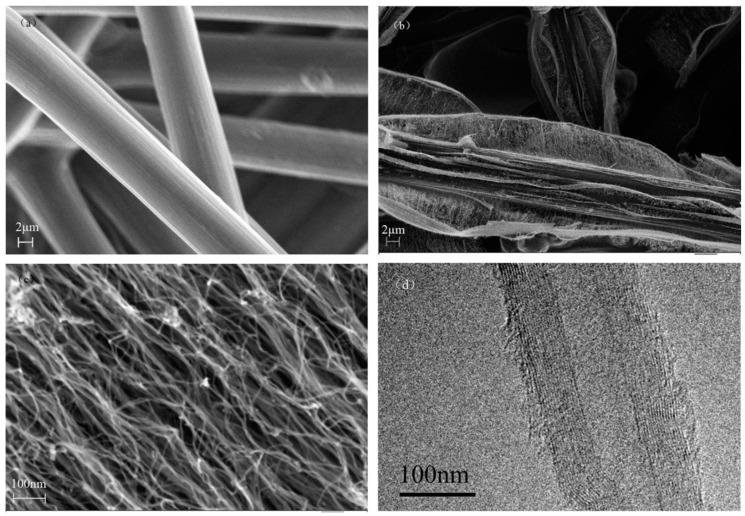
SEM micrographs of (**a**) before and (**b**) after growth of VACNT on CF paper coated with catalytic film, whereas (**c**) presents the VACNT at higher magnification; (**d**) TEM micrograph of the produced CNT. Reprinted with permission from Zhang, Journal of Physics and Chemistry of Solids; Ref. [123] copyright 2017, Elsevier.

**Figure 26:**
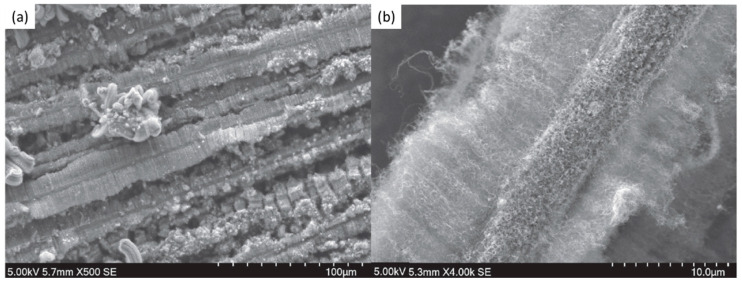
(**a**) CVD-grown VACNT across thin-ply CF tape; (**b**) idem at higher magnification. Reproduced with permission from Russello, Composite Structures; Ref. [125] copyright 2018, Elsevier.

Finally, several studies showed the growth of radially aligned CNT or VACNT on desized or/and surface-modified CF via CVD [120,121,122,123,124,125]. Li et al. (2015) [122] performed adsorption of polymeric functional coating Poly(styrene-alt-[dipotassium maleate]) (K-PSMA) on sized CF to introduce surface potassium ions available for Fe catalyst (0.05 M Fe(NO_3_)_3_·9H_2_O in isopropanol) adhesion and avoid the interaction/diffusion of the later with-/-in the CF structure. This concept of ‘direct’ CNT synthesis on CF resulted only in homogenous growth of low density short radially aligned CNT on the CF circumference, the later aligned in dense unidirectional filament yarn (Figure 23). Contrary to previous studies (cited therein), the authors performed longitudinal tensile and interface shear strength testing to show that the mechanical properties of the used CF was retained and further improved with the synthesis of radially aligned CNT. Another point influencing the suppressed thermal, but also, catalytic degradation of the CF structure is the performance of the CVD growth of CNT at temperature as low as 480 °C. Contrary to the production of radially aligned CNT (Figure 23) [122], Rahmanian et al. (2013) [120] and Zhang et al. (2017) [123] performed growth of VACNT ribbons, evidenced in double or multiple rows, on the CF circumference (as shown in Figure 24c and Figure 25b, respectively). To directly synthesize VACNT via CVD [120] or PECVD [123], both groups performed chemical activation pre-treatment to enhance the anchoring of catalyst nanoparticles on CF. The CFs were oxidized through immersion in 65% HNO_3_ [120] or 2M HNO_3_ [123] and subsequently impregnated with dense Fe catalyst coating (0.0001M Fe(NO_3_)_3_·9H_2_O in acetone [120] or Fe(NO_3_)_3_ in ethanol [123]). At an elevated temperature of 250 °C, a calcination process followed the coating process. Moreover, the mild oxidation process created carbon-oxygen functional groups (carbonyl and carboxylic groups), previously observed to enhance the formation of carbon-metal bonds [5], but also introduced improved surface roughness by etching-out present amorphous carbon zones (Figure 24a,b). The developed surface roughness further influenced the homogeneous deposition of catalyst nanoparticles for the growth of VACNT (Figure 24c). Thus, performed TEM analyses show the presence of multi-wall CNT (MWCNT), with an outer diameter of 20 nm and an inner diameter of 5 nm (Figure 24d and Figure 25d). Additionally, the developed specific surface area is considered advantageous for using the final composites in electro-chemical applications. Details of CF modification, impregnation of catalyst and CVD growth of VACNT are given in Table 8. Noteworthy, Russello et al. (2018) [125] present direct growth of VACNT on spread CF tow tapes via pure-thermal CVD (details of catalyst deposition and CVD growth of VACNT are given in Table 8). Again, 0.2 M Fe(NO_3_)_3_·9H_2_O in isopropanol was adsorbed and dried on the surface of the CF prior to the CVD process, yet without the pre-adsorption of any polymeric functional coating and/the CVD process was performed at elevated temperature of 700 °C. The study presents the growth of short, VACNT (2–10 µm) with high density that resulted in the formation of VACNT ribbons (Figure 26). Moreover, the CNT growth process was stopped before the catalyst inactivation, thus, prolonged CVD process might result in VACNT with superior height, but also, influence structural degradation of the CF substrate. The ‘direct’ growth of 1–2 µm tall VACNT on CF yielded to composites with through-thickness electric conductivity of 0.11–0.22 S cm^−1^, respectively [125].

Though achievable, the growth of VACNT with the suggested methods is highly restrictive and cannot be considered viable for tuneable growth of VACNT on CF due to:

1. The properties of some of the examined carbonaceous materials (ex., highly graphitized materials with a precise ratio of 85:15 sp^2^:sp^3^ hybridization [32], oxidative plasma-treated HOPG [48], or GP [49]) do not reflect the structural properties of commercially used carbon substrates (as CF, mostly ex-PAN [126,127,128]) available for large-scale industrial use and applications.
2. The composite properties stability, which depends on the controllable growth of VACNT (CNT length/diameter/density), is challenging to achieve when direct growth is performed on CF substrates.
3. Presented pre-treatments, such as mild oxidation/activation, of the CF before the growth of VACNT should influence the CF’s structural and physical properties.

Accordingly, this subsection is only a critical review of the ‘direct’ growth of VACNT on carbon substrates without the presence of intermediate DBL. Yet, the derived information does not justify the direct use of evidenced methods for the controlled growth of highly dense and long VACNT on CF. Nonetheless, the recovered information can be seen as an excellent bibliographic backup for advanced direct VACNT growth on carbon substrates within extended time framings. Within the following subsection, different methods are thus deeply explored for the growth of VACNT on CF with suitable conducting DBL and the development of new composites.

### 3.2. Deposition of Conducting DBL for Growth of VACNT on Carbon Substrates, with a Focus on CF Substrates

#### 3.2.1. Via Chemical Vapor Deposition (CVD)

**Figure 27:**
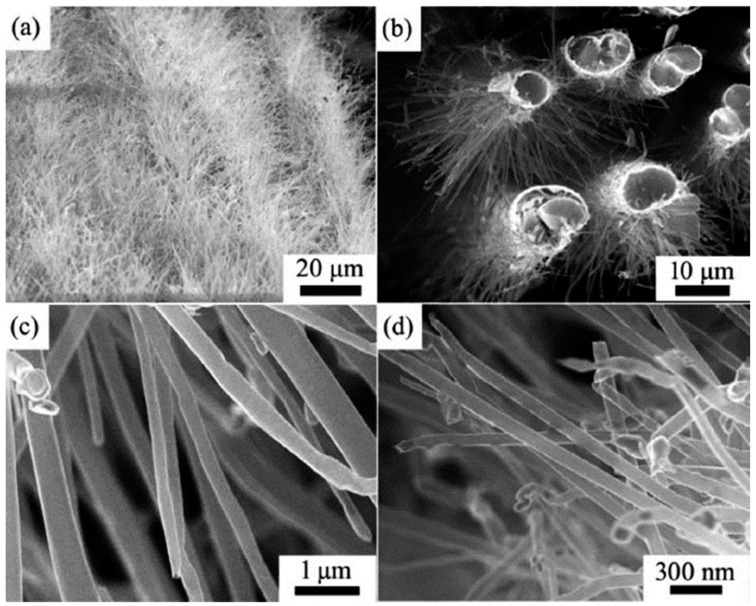
SEM micrographs of radially aligned CNT grown on PyC covered CF: (**a**) Top-view and (**b**) cross-section of CF, whereas (**c**,**d**) are magnifications of zones with CNT. Reprinted with permission from Sun, Vacuum; Ref. [45] copyright 2019, Elsevier.

In the last decade, radially aligned CNT has been grown on pyrolytic carbon (PyC) DBL deposited on CF materials by CVD [45,129,130]. The predeposition of PyC protects the CF surface properties by suppressing the interactions with the catalyst and sub-layer diffusion of the latter. The PyC is a promising DBL due to its compatibility with commercial CF, derived from PAN or pitch. Moreover, this kind of DBL resulted in the advanced growth of straight CNT with higher purity and length when compared to the direct growth of CNT over CF (Figure 27). The examined CVD protocols and composite properties are summarized in Table 9. The radially aligned CNT on PyC DBL positively affected the final composites’ oxidation resistance and mechanical properties. The mechanical properties improvements result from the effective transfer of the mechanical stress from the matrix to the CFs via the intermediate presence of CNT and the PyC [45,130]. Compared to neat C/C composites, calefactive oxidation tests show superior oxidation resistance of C/C composites with grown aligned CNT [129]. However, the low density and high diameter CNT obtained through this approach still demand further research before reaching an advanced technological readiness level compared to VACNT obtained on metal oxide, insulating, DBL [36,43,44,95,96,97,98,99]. Moreover, the high-temperature CVD deposition of the PyC (above 1000 °C) is a disadvantage for the use of the presented DBL that should influence the final cost and commercialization of potential CF-VACNT composites.

**Figure 28:**
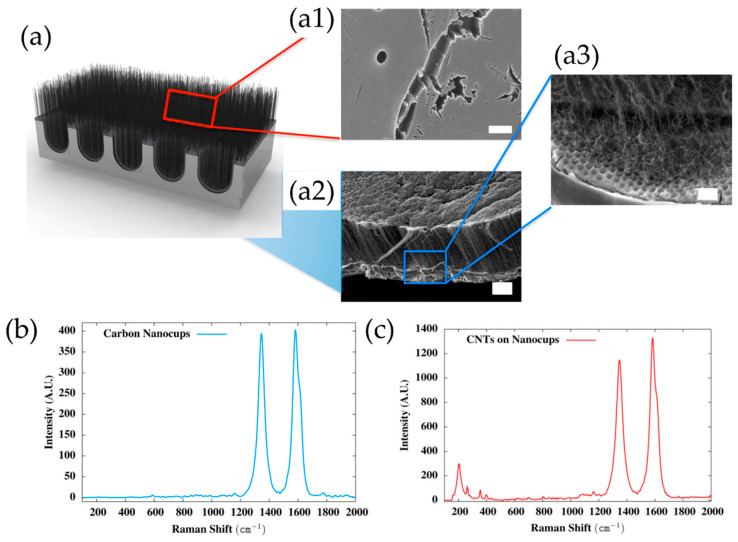
(**a**) Matching schematic representation of SEM micrographs of carbon nanocups with grown VACNT seen from the top (**a1**) and (**a2**) side view with (**a3**) presenting a zoom on the nanaocups-CNT interphase. The scale bars are 200 μm, 4 μm, and 400 nm, respectively; (**b**) Raman spectra recorded from neat nanocups sample, where the A_D1_/A_G_ ratio is 1.22; (**c**) Raman spectrum of VACNT−nanocups composite with A_D1_/A_G_ ratio of 1.08 and occurring radial breathing mode due to single-wall CNT. Reprinted with permission from Hahm et al. (2012); Ref. [92] Copyright 2012 American Chemical Society.

Hahm et al. (2012) [92] have also investigated the use of CVD for both synthesis of a graphitic substrate and the growth of VACNT. An anodized aluminium oxide template with nanocups morphology was also used to critically enlarge the specific surface area of the graphitic substrate, i.e., the final composite. Following the CVD of the graphitic substrate, PVD of a 1.5 nm Fe catalyst layer was performed before a second CVD process to grow VACNT (Details given in Table 9). Resultant, dense carpets of single- (Figure 28c), double- or multi-walled CNT with 5–10 µm length were grown on the graphitic substrate to produce a 3-D hybrid composite, tested as a supercapacitor (Figure 28(a, a1–3)). The thickness of the synthesized graphitic substrate (the graphitic nanocups) was not mentioned, and the use of Raman spectra presented a high degree of structural disorder, based on the strong intensity of the D band (1364 cm^−1^) (Figure 28b). Contradictory to the latter, Cartwright et al. (2014) [32] confirmed the use of substrate rich in sp^3^ carbon as inadequate for forming a homogenous network of active Fe catalyst nanoparticles and further growth of VACNT. Though not stated, the anodized aluminium oxide template (present during the PVD of Fe catalyst and the CVD processes) might have affected the observed Fe catalyst surface stabilization and growth of VACNT. Finally, the VACNT−carbon nanocups-based supercapacitor exhibited 50% higher specific capacitance (0.6 mF/cm^2^) than the reference carbon nanocups one. The increase in the specific capacitance was attributed to the larger surface area and hybrid 3-D morphology of the VACNT−carbon nanocups-based supercapacitor. Cyclic stability tests showed stable capacitance even up to 10,000 charge/discharge cycles. More important, the electrochemical impedance spectra measurements presented a low equivalent series resistance of 23 Ω between the VACNT−carbon nanocups electrode and gold current collector. 

The CVD deposition of hybrid metal oxide/nitride, graphitic, and other promising conducting DBL has yet to be investigated for the growth of VACNT on carbonaceous substrates. To the best of our knowledge, no other studies were identified to use CVD to achieve deposition of conducting DBL and the growth of VACNT on carbon materials.

#### 3.2.2. Via Physical Vapor Deposition (PVD)

PVD has been used to deposition DBL further used in at least two methods for the CVD growth of VACNTs on CF.

**Figure 29:**
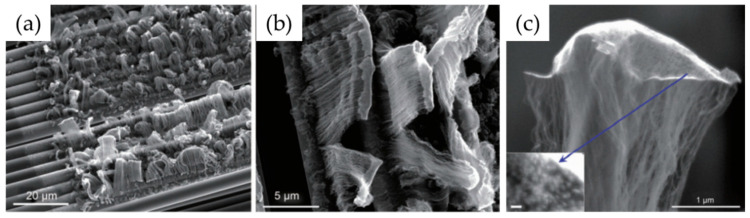
(**a**) SEM micrograph of ‘*Odako*’ grown SWNTs on CFs. (**b**,**c**) are zoom of (**a**) to confirm the presence of detached alumina flakes with insert in (**c**) showing Fe catalyst that supports tip-growth of VACNT. Scale bar in (**c**) of 50 nm. Reported after [131].

Though not a real conducting interface between a CF and VACNT, yet, playing the role of DBL for the preservation of surface-active Fe catalyst nanoparticles, researchers have demonstrated the use of appropriate capping top layer via the ‘*Odako*’ method [57,131,132,133,134,135,136]. The ‘*Odako*’ VACNT growth of VACNT is performed with a distinctive CVD technique for ultra-fast detachment and fragmentation of the extremely thin capping layer with incorporated catalyst nanoparticles. This method is also discussed to allow a direct CNT covalent interface with a carbon substrate [131] (Figure 29). Usually, an alumina capping layer (of a few nanometers) is preferred when a Fe catalyst is used to grow VACNT. The Fe catalyst is immobilized on the alumina layer due to interfacial reactions yet kept active and uncovered to develop the formation and growth of CNT [115]. 

The presented ‘*Odako*’ method is advantageous compared to others, except for the direct growth of VACNT on carbon substrate, since in both ways, the VACNT are anchored on a carbon substrate via covalent carbon-carbon bonds. The latter is vital for optimal adhesion and electrical contact between the carbon substrate and the VACNT. Using suitable electrolyte and electrochemical test conditions, ‘*Odako*’ achieved VACNT/carbon (graphite, graphene) composites have been examined as excellent capacitors [57,132,133,136]. Although highly innovative and beneficial, there are a few criteria for the performance of this method, such as:

- Use of special engineering approaches to perform precise deposition of a few nanometer layers of alumina with specific physical density and oxidation state;
- To grow VACNT on the catalyst lower layer, a particular joule heating or similar CVD system should be used to stress crack the alumina top layer into small fragments under the sharp increase in temperature at the rate of 300 °C min^−1^. Thus, deviations in the capping layer thickness and the CVD heating rate can disturb the activation of the Fe catalyst and the VACNT growth;
- The performance of the ‘*Odako*’ method demands an additional plasma etching process to remove the catalyst-alumina layer standing on the top of the VACNT.

All the above-numbered are some points of the ‘*Odako*’ method that restrains and limits the immediate use of this technique on an industrial scale. In addition, one should further examine the commercial viability and the cost of this production method of carbon substrate-VACNT hybrids. The recovered information should be further compared with the composite production using conducting DBL (between the CF and the VACNT) with an advanced technological readiness level. Inline, the ‘*Odako*’ method is herein referenced as future long-term solutions to be developed for commercial growth of VACNT on CF/carbon substrates.

Contrary to the mentioned ‘*Odako*’ method, PVD of conducting DBL is widely investigated for further growth of VACNT via conventional pure thermal CVD, seen as an easy-to-industrialize process with advanced technological readiness level. PVD predeposition of 20 nm Ni (as both DBL and catalyst) has been shown to support fuzzy CNT on CF via thermal CVD at temperatures as low as 550 °C [22]. The results present Ni as a DBL/catalyst for the development of a fuzzy CNT network that boosted the mechanical stiffness and further improved the electrical conductivity of the final composites. However, VACNT on CF have not been reported with the examined PVD deposited Ni DBL/catalyst and CVD process. Another pioneering study [137] presents the growth of short VACNT on CF with PVD pre-deposition of Ti DBL and again Ni catalyst top-layer. Moreover, the study endorses the formerly observed failure of lone Ni DBL/catalyst to produce VACNT without the presence of 5 nm Ti under-layer due to the extensive reaction between the CF surface and the catalyst. Unfortunately, the effect of the Ti DBL cannot be clearly justified due to the applied electric field during the performed plasma-enhanced CVD that influences the directional growth of VACNT (as seen in [48,50]). Fang et al. (2017) have also investigated the use of 70 nm Ti DBL to support 12 nm Ni catalyst and the subsequent growth of VACNT via pure-thermal CVD at a high temperature of 850 °C [80]. As a result, only fuzzy CNT, of 100 nm mean diameter and 2–3 µm length, were observed on the surface-modified CFs even after a CVD duration of 30 min. The latter confirms the inability of a Ti DBL to support the formation of a few nanometer metal nanoparticles and retain an active Ni catalyst to achieve VACNT. Finally, the growth of VACNT on CF cannot be effectively performed only with Ni DBL/catalyst due to the metal diffusion within the CF structure. In addition, the use of Ti DBL/Ni catalyst solution was examined effective only via the plasma-enhanced CVD method. Thus, the latter was not achieved (or should be reevaluated) via pure-thermal CVD.

**Figure 30:**
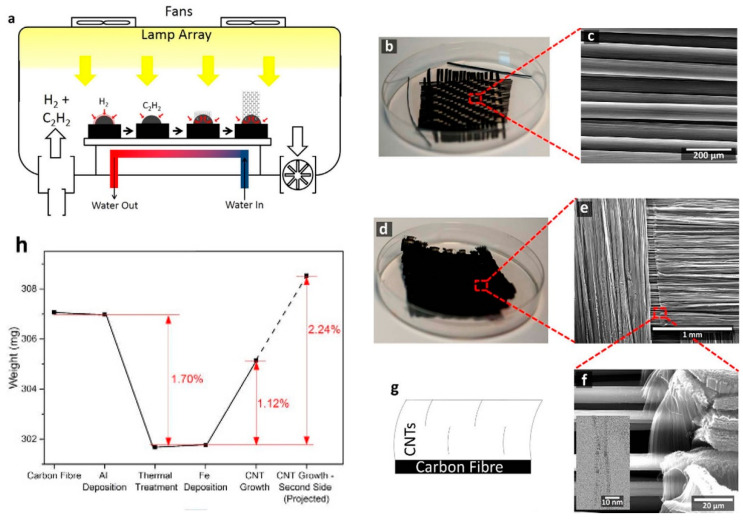
(**a**) Schematic representation of PTCVD for the growth of VACNT; (**b**) Digital image of as-received CF cloth with (**c**) SEM micrograph of aligned CF; (**d**) Digital image of VACNT/CF cloth composite material with (**e**,**f**) different magnification of SEM images where (**f**) presents an insert of STEM micrograph of the examined multi-wall (MW)CNT; (**g**,**h**) Figurative representation of the weight change after each consecutive step in the twin-side VACNT/CF cloth composite growth. Modified after [4].

While no CNT were observed on bear CFs, bundles of aligned multi-walled carbon nanotubes (MWCNT) were synthesized by floating catalyst CVD on CF paper with predeposited 10 nm Al DBL [46]. Once again, the exposed presence of native alumina was presented as a crucial factor for stabilizing the Fe catalyst nanoparticles and the improved growth of high-density VACNT. Yet, the thickness of 10 nm of Al DBL was not observed as sufficient to form a continuous and dense network of Fe nanoparticles for self-sustaining VACNT bundles that collapsed with the increase in the CNT length (observed at around 20 µm). Another recent study shows the beneficial effect of monometallic Al DBL on the growth of VACNT on CF substrates. Pozegic et al. (2016) [4] presented CVD growth of VACNT with improved density, length, and alignment on CFs than previously reported study [46]. The VACNT properties are improved mainly due to 35 nm Al DBL that minimized the interaction between the substrate and the catalyst. Previously, Pozegic et al. (2014) [138] confirmed the incompetence of PVD deposited Fe catalyst alone to grow VACNT via the same CVD process, producing only scattered fuzzy CNT on the CF surface. Figure 30 presents the scheme of used photo-thermal CVD, morphological details of the CF prior/post VACNT growth, and the variation of the CF cloth material passing from one preparation phase to the next one. Interesting to note is the PDV deposition of Al DBL without desizing the CF surface polymer layer that has not been previously reported and did not influence the effective growth of VACNT. Once more, the growth zone of VACNT was imposed from the PVD of the Al DBL, only present on the outer CF cloth surface. Detailed information regarding the synthesis conditions and the CNT properties can be seen in Table 9. As previously mentioned, Al DBL formed a native oxide top-layer during the air transfer and catalyst annealing process. The latter confirmed by numerous studies to improve the formation and anchoring of Fe catalyst nanoparticles for promoted growth of VACNT [60]. The formation of this top-layer oxide can further increase the electrical resistance of an Al DBL layer by severe order of magnitude [34], thus influencing its initial role as conducting DBL. However, Pozegic et al. (2016) [4] reported improved overall composite conductivity (electrical and thermal) by a few hundred percent due to the low (but sufficient for the purpose) thickness of the DBL layer, which retains its conducting purpose. In addition, the beneficial use of photo-thermal CVD (PTCVD) is shown as an easily scalable/controlled optical top-heating method for the growth of VACNT. Thus, the use of PTCVD in combination with appropriate conducting DBL, with good optical reflectance of infra-red/ultra-violet radiation, is presented to boost the thermal decomposition of the carbon precursor on the irradiated catalyst nanoparticles and simultaneously protect the thermal degradation of the used substrate.

**Figure 31:**
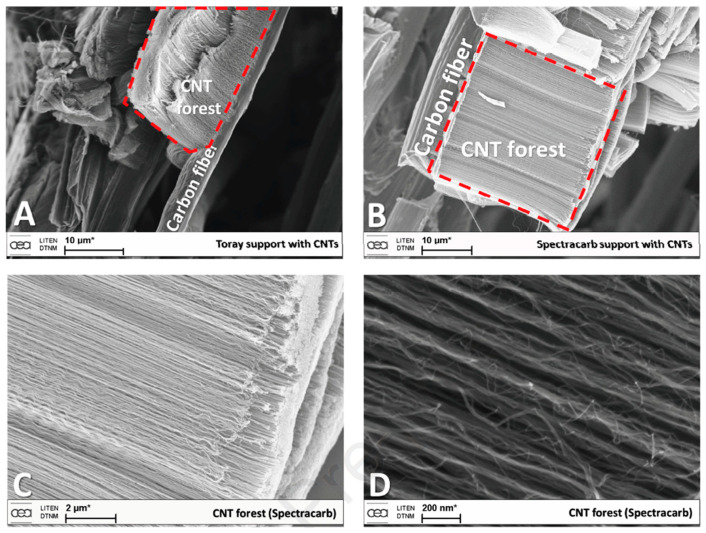
SEM micrographs of VACNT grown on CF of commercial GDLs with PVD pre-deposited TiAl/Fe DBL/catalyst layer. (**A**) low magnification of Toray GDL. (**B**–**D**) low, high, and highest magnification of Spectracarb GDL with grown VACNT, respectively. Reprinted with permission from Fontana, Carbon; Ref. [47] copyright 2020, Elsevier.

**Figure 32:**
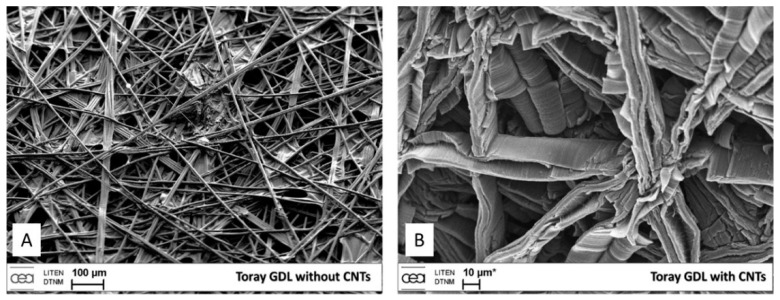
Top–view SEM micrographs of (**A**) As-received Toray GDL and (**B**) VACNT/Toray GDL. Reprinted with permission from Fontana, Carbon; Ref. [47] copyright 2020, Elsevier.

Furthermore, a more recent study by Fontana et al. (2020) [47] describes an effective way to grow dense VACNT on CF gas diffusion layers (GDL) via hot-filament CVD at temperatures as low as 500 °C (Figure 31). The key to the successful growth of VACNT was the use of PVD deposited diffusion barrier bi-layer of Ti/Al metal alloy with a Fe catalyst top-layer. Primarily, the Ti under-layer was stated (yet not tested in the study) to promote the DBL adhesion with the CF surface. Secondly, Ti alloy with Al (forming titanium aluminide—TiAl) was stipulated to suppress the strong surface oxidation of the Al layer that preserved its electrical conductivity properties. Moreover, the authors validate the importance of the Al upper layer for the successful annealing of the Fe catalyst layer, i.e., the formation of a dense network of active Fe catalyst nanoparticles for the growth of VACNT. In addition, the morphology of the produced VACNT was solely correlated with the properties of the PVD deposited DBL/catalyst and the CVD process, excluding any influence from the CF surface morphology and/or chemistry. Due to the highly directive PVD deposition of the DBL/catalyst coating, the growth of the VACNT was also oriented and confirmed on the PVD exposed surface of the GDL (Figure 32). In contrast, the same was not observed on the opposite side and the substrate interior. Details of the PVD deposition and the CVD protocol for the growth of the VACNT can be found in Table 9. Finally, the authors presented VACNT modified GDL as a cathode to improve the overall fuel cell performances up to 1.7 A/cm^2^ (current density improvement up to 30% at 0.75 V or 50% efficiency for a fuel cell) in comparison with up-to-date best state-of-the-art GDL.

## 4. Conclusions

Future developments require rapid technological advancements in various material applications. The use/growth of VACNT on different substrates through the predeposition of conducting DBL is a promising approach, with an intense scientific exploration towards the improvement of existing and the development of new composites. As a recent branch of carbon composites, CF-VACNT exhibits excellent potential to go beyond existing limits. Three-dimensional flexible CF-VACNT composites are being extensively explored, owing to their unique morphological and physico-chemical properties. Recent progress on the introduction of DBL with improved conducting properties should meet the requirements needed for advanced construction composites, state-of-the-art wearable sensors or power devices, and many other applications.

In this review, we summarized and discussed various deposition/synthesis methods and techniques used to deposit conducting DBL and grow VACNT on flat or 3-D substrates of different natures, with a focus on fibrous carbons. The parameters/conditions for each of these methods and techniques and the nature/properties of the deposited DBL/grown VACNTs have been shown to affect the final composite and its performances compared to rivals or reference materials. The post-deposition evolution of a conducting DBL, CNT catalyst diffusion/deactivation, or the process condition restrictions have been generally determined as common bottleneck points towards robust fabrication technology. 

Overall, the conducting DBL plays a significant role in both the growth of tunable VACNT and the final physico-chemical properties of the composite. Thus, the conducting coupling of a concerned substrate and VACNT with appropriate DBL is a challenging topic under examination, aiming at the cost-effective mass-production of novel high-performant materials.

## Figures and Tables

**Table 1:** Details of used substrate, lone Al DBL (deposited via PVD), and VACNT growth conditions and properties.

Substrate	DBL via PVD/PVD Conditions for DBL/Catalyst	Process of CVD/Carbon Source	Carrier or Carbon Source Gas: Flow Rate/Mixture (m^3^ s^−1^, Unless Otherwise Specified)	Pressure (Pa)/Temperature (K)/Reaction Duration (min)	CNT Average Diameter (nm)	CNT Length (µm)	Density of VACNT (Tubes cm^−2^)	DBL/VACNT/Composite Properties	Reference
Si coated with native SiO_2_ or additional 300 nm thick SiO_2_ top-layer	**10 nm Al DBL via magnetron sputtering**/Chamber pressure (5 × 10^−8^ Pa); Operating gas pressure (7 × 10^−1^ Pa); Operating gas Ar at 2 Pa; Operating temperature: 293.15, 320.15, 373.15, 473.15 K/0.5 nm Ni catalyst deposited via electron beam evaporation at a rate of 0.01 nm s^−1^ (post-transfer in air)	**Alcohol assisted thermalCVD (tube furnace)**/C_2_H_5_OH and H_2_	- CNT Synthesis: H_2_:C_2_H_5_OH:Ar = 1.3 × 10^−7^: 1.2 × 10^−5^: 2 × 10^−5^	P_atm_/1073/**Annealing (5); Synthesis (20)**	7	30–40	-	- Use of metal alloy as DBL/catalyst layer - Growth of single-wall VACNT	[65]
Si coated with 4 nm native SiO_2_	**18 nm Al DBL via electron beam evaporation**/Chamber pressure (0.0007 Pa); Operating temperature: 293.15 K; Deposition rate: 0.1, 0.2, 0.3 and 0.4 Å s^−1^/2 nm Fe catalyst deposited via electron beam evaporation at 0.1 Å s^−1^ (break of vaccum before the Fe deposition)	**Water-assisted CVD**/Ar, H_2_O vapors and C_2_H_2_	-	3.23/973/**Annealing (6); Synthesis (10)**	- CNT inner diameter: 4–9 nm (0.4–0.1 Å s^−1^) - Number of walls in CNT: 6–8 (0.4–0.1 Å s^−1^)	78–150 (0.1–0.4 Å s^−1^)	-	- Growth of multi-walled VACNT	[68]
Si coated with SiO_2_ with 300 nm thickness	**30 nm Al DBL via magnetron sputtering**/Operating temperature: 293.15 K/0.5 nm Fe catalyst deposited via electron beam evaporation (post transfer in air)	**Thermal CVD (quartz tube reactor)**/C_2_H_2_ and He	- Annealing: He 20 slm; - CNT Synthesis: He 20 slm; C_2_H_2_ 6.7 × 10^−6^;	P_atm_/1053/**Annealing (20); Synthesis (15)**	-	1330	3 × 10^10^	- 5 cm long CNT yarns from twisted CNT.	[66]

**Table 2:** Details of used substrate, lone or modified Al DBL (deposited via PVD), and VACNT growth conditions and properties.

Substrate	DBL via PVD/PVD Conditions for DBL/Catalyst	Process of CVD/Carbon Source	Carrier or Carbon Source Gas: Flow Rate/Mixture (m^3^ s^−1^, Unless Otherwise Specified)	Pressure (Pa)/Temperature (K)/Reaction Duration (min)	CNT Average Diameter (nm)	CNT Length (µm)	Density of VACNT (tubes cm^−2^)	DBL/VACNT/Composite Properties	Reference
Poly Si wafer	**Co-Al (99.5%)** /-/1 nm of Fe catalyst film	**pure-thermal**/C_2_H_2_, H_2_, and He	Ratio data: C_2_H_2_:H_2_:He = 1:5:5	100/853/-	~4–5	2–30	Up to 2.5 × 10^12^	- Growth of double-/triple--walled VACNT - Electrical conductivity measured by AFM tip on CNT carpet with a contact force of 100 µN or 1N, measured minimal resistance of ~20 Ω or ~2.1 Ω, respectively. The resistance linearly increased with the CNT length.	[67]
400 µm thick Cu foils (99.99% Cu, Vortex metals, Inc., Warrensville Heights, OH, USA)	**5–10–15–30 nm Al DBL via RF sputtering (DV-502A, Denton Co., Cardiff, UK)**/-/Catalyst introduced during CVD: ferrocenne/m-xylene (10 kg m^−3^)	**Floating catalyst CVD (quartz tube reactor)**/Carbon source: ferrocene/m-xylene (10 kg m^−3^) with Ar and H_2_ as carrier gas	- CNT Synthesis: H_2_ 8.3 × 10^−7^; C_2_H_2_ 1.6 × 10^−6^;	P_atm_/1023/**Annealing (20); Synthesis (15)**	10–100	30–50	-	- Interfacial adhesion (pull-off tests) tests: around 0.2 to 0.4 MPa as for 10 to 30 nm Al DBL composites, respectively.	[51]
25 µm thick Cu foils (99.8% Cu, Alfa Aesar, No: 046365.RH)	**6 nm Al DBL via DC magnetron sputtering**/Chamber pressure (1 × 10^−6^ Pa); Operating gas pressure (3.5 × 10^−3^ Pa); Operating gas Ar; DBL deposition rate: 0.022 nm s^−1^/Successive deposition of 0.4 nm Fe catalyst; Deposition rate: 0.018 nm s^−1^ + additional deposition of 0.4 nm Al top layer	**Cold-wall CVD**/C_2_H_2_ and H_2_	- Annealing: H_2_ 8.3 × 10^−6^; - CNT Synthesis: 98% H_2_/2% C_2_H_2_ = 8.3 × 10^−6^;	(1.5 × 103)/Annealing (953); Synthesis (1033)/**Annealing (1.5); Synthesis (0.5)**	1.9	9.2	2.2 × 10^12^	- Bi-modal SWCNT diameter distribution - Resistivity of SWCNT-Al DBL- Cu composite: 60–80 Ohms	[73]
Conducting brass (Cu-Zn alloys), copper, stainless steel, Inconel® 600, silicon, and alumina	**2–5–10–20 nm Al DBL via RF sputtering**/-/1 nm Fe catalyst deposited via electron beam evaporation (air introduced between the Al DBL and the Fe PVD steps)	**Cold-wall low-pressure CVD**/C_2_H_2_ and H_2_	- Annealing: H_2_ 1 × 10^−5^; - CNT Synthesis: H_2_ ?/C_2_H_2_ ?	(5 × 10^3^)/Annealing (973); Synthesis (933)/**Annealing (5); Synthesis (10)**	7	46–312 (depen-ending on DBL thickness and susbtrate type)	-	- Growth of multi-walled VACNT; - Total electric resistance ranging below 10 Ω; - Specific capacitances were measured to be 8.1 ± 0.6, 7.9 ± 0.4, and 10.0 ± 0.4 F g^−1^ for VACNT on Inconel^®^ 600, Cu, and stainless steel, respectively, (Cyclic voltammetry measurements in aqueous KOH electrolytes)	[72]
TiSi	**2 nm Ni—1 nm Al—2 nm Ni was deposited by electron beam** **evaporation**/Chamber pressure (4 × 10^−5^ Pa); deposition rate: 0.2 Å s^−1^	**200 W Plasma-enhanced CVD**/CH_4_ and H_2_	- Annealing: H_2_:N_2_ = 5 × 10^−8^: 1.6 × 10^−8^; - CNT Synthesis: H_2_:N_2_ = 5 × 10^−8^: 1.6 × 10^−8^ + 35 sccm CH_4_	370/923/**Annealing (10); Synthesis (1)**	~43.6	4.5	2 × 10^10^	- Growth of thick multi-walled VACNT; - Achieved Ohmic contact between the Ti silicide and the VACNT with a total resistance of 540 Ω.	[91]

**Table 3:** Details of used substrate, metal or metal nitrides DBL without the use of Al top-layer (deposited via PVD), and VACNT growth conditions and properties.

Substrate	DBL via PVD/PVD Conditions for DBL/Catalyst	Process of CVD/Carbon source	Carrier or Carbon Source Gas: Flow Rate/Mixture (m^3^ s^−1^)	Pressure (Pa)/Temperature (K)/Reaction Duration (min)	CNT Average Diameter (nm)	CNT Length (µm)	Density of VACNT (tubes cm^−2^)	DBL/VACNT/Composite Properties	Reference
Oxidized Si/SiO_2_ coupons	**30 nm Ta via electron-beam evaporation**/Operating gas pressure (3.5 × 10^−4^–3.5 × 10^−5^ Pa)/Electron-beam evaporation of 5 nm Fe catalyst film	**Fast-heat 3 zone thermal**/C_2_H_4_	Ar (3.3 × 10^−6^): C_2_H_4_ (2.5 × 10^−6^): H_2_ (6.6 × 10^−6^)	P_atm_/Furnace zone I and II -annealing step (1023); Zone III—CNT growth (748)/**(45)**	~22	~2	-	- Electrical conductivity measured by AFM tip on contact with CNT carpet, measured resistance ~35 kΩ	[62]
Si wafer	**130 nm TaN via conventional radio frequency (RF:400 W) magnetron sputtering**/Operating gases N2 (3 × 10^−8^–3 × 10^−7^ m^3^ s^−1^) + Ar (1.7 × 10^−6^ m^3^ s^−1^); Operating gas pressure (5 Pa)/2.5% ferrocene in toluene	**aerosol-assisted catalytic**/toluene	Ar (5 × 10^−5^)	P_atm_/(1123)/**(12)**	~60–150	~40–150	-	-	[77]
Si wafer	**Post-deposition 60 nm and post-annealing 60–240 nm TaN thickness via highly pulsed power magnetron sputtering**/Operating gases (N_2_+Ar)/5% ferrocene in toluene	**aerosol-assisted catalytic**/toluene	Ar	P_atm_/(1123)/**(15)**	~40–110	~45–195	-	-	[78]
Si coated with SiO_2_	**25 nm Co-Zr-N-(O) DBL via DC magnetron sputtering with Zr target**/Chamber pressure (10^−3^ Pa); Operating gas pressure (0.7 Pa); Operating gas Ar/N2 ratio: 10/80 vol. %/Co target (final atomic concentration 30–35%)	**Plasma System in CVD mode without plasma**/C_2_H_2_ and H_2_	- CNT Synthesis: NH_3_:C_2_H_2_:Ar = 1.6 × 10^−8^: 1.6 × 10^−8^: 5 × 10^−8^	-/783/**Annealing (/); Synthesis (10)**	7	30–40	-	- Use of metal alloy as DBL/catalyst layer - Growth of multi-walled VACNT	[112]

**Table 4:** Details of used substrate, metal nitrides/silicide-nitrides DBL without using Al top-layer (deposited via PVD), and VACNT growth conditions and properties.

Substrate	DBL via PVD/PVD Conditions for DBL/Catalyst	Process of CVD/Carbon Source	Carrier or Carbon Source Gas: Flow Rate/Mixture (m^3^ s^−1^)	Pressure (Pa)/Temperature (K)/Reaction Duration (min)	CNT Average Diameter (nm)	CNT Length (µm)	Density of VACNT (tubes cm^−2^)	DBL/VACNT/Composite Properties	Reference
Laminated copper sheets	**20 nm TiN via magnetron sputtering**/Operating gas N_2_+Ar; Operating temperature: 293 K/8.76% ferrocene in toluene as catalyst/carbon source (injection rate: 5.6 cm^3^ h^−1^ in preheated evaporator at 453 K	**Pure-thermal CVD (tube reactor)**/-	- Annealing and CNT Synthesis: Ar (4.2 × 10^−5^): H_2_ (4.2 × 10^−6^)	≥P_atm_/(973)/**Annealing (10); Synthesis (20)**		20–30		- Growth of bamboo-like multi-walled VACNT with intence structural defects;	[83]
P-type silicon wafers (100) El-CAT Inc., Ridgefield Park, NJ, USA	**200 nm TiN via Dual Ion Beam Sputtering (DIBS) with TiN target (99.5% purity)**/Operating gas N_2_+Ar; Operating temperature: 493 K; Duration 240 min/Mo(CH_3_COO)_2_ (0.04% p/v) and Co(CH_3_COO)_2_·4H_2_O (0.02% p/v) in C_2_H_5_OH via dip-coating.	**alcohol catalytic**/N_2_ flow saturated with ethanol:water (99.5:0.5 *v*/*v*)	- Annealing: Ar (4 × 10^−6^); - CNT Synthesis: N_2_ (4 × 10^−6^): H_2_ (1.3 × 10^−6^): EtOH (8.3 × 10^−6^)	P_atm_/(1073, 30 K min^−1^)/**Annealing (10); Synthesis (20)**	~3–5	~8	-	- Field emission performance under therein given conditions	[88]
Si coated with 200 nm thermal SiO_2_	**50 nm TiSiN, or TiN DBL via sputtering**/-/1 (for TiSiN), or 5 nm (for TiN) Fe catalyst deposited via evaporation (air introduced between the DBL and the Fe PVD steps)	**Hot-wall CVD (quartz tube reactor)**/C_2_H_2_ and H_2_	- Annealing:Ar:H_2_ = 1.6 × 10^−5^ : 8.3 × 10^−6^; - CNT Synthesis: C_2_H_2_:Ar:H_2_ = 1.6 × 10^−7^:1.6 × 10^−5^: 8.3 × 10^−6^;	P_atm_/973, 100 K min^−1^/**Annealing (5); Synthesis (15)**	~5 (TiSiN); ~6–9 (TiN)	~200–300	~10^12^ (TiSiN); ~10^10^ (TiN)	- Growth of multi-walled VACNT - Air current–voltage measurements with two-terminal probe station yield resistance values of 0.8 and 3.4 kΩ for TiSiN and TiN, respectively.	[89]

**Table 5:** Details of used substrate, metal or metal nitrides/silicide-nitrides DBL using Al top-layer (deposited via PVD), and VACNT growth conditions and properties.

Substrate	DBL via PVD/PVD Conditions for DBL/Catalyst	Process of CVD/Carbon Source	Carrier or Carbon Source Gas: Flow Rate/Mixture (m^3^ s^−1^)	Pressure (Pa)/Temperature (K)/Reaction Duration (min)	CNT Average Diameter (nm)	CNT Length (µm)	Density of VACNT (tubes cm^−2^)	DBL/VACNT/Composite Properties	Reference
**Si coated with native SiO_2_**	**50 nm TiSiN using MaTecK GmbH TiSiN target and 6 keV Ar ions**/-/0.4 nm Fe catalyst + 0.1 nm Al (top-cover) deposited via magnetron sputtering in 0.35 Pa of Ar at 20 W (air transfer between the PVD and CVD steps)	**Pure-thermal CVD (tube reactor)**/C_2_H_2_ and H_2_	- Annealing: Ar:H_2_ = 1.6 × 10^−5^:8.3 × 10^−6^; - CNT Synthesis: C_2_H_2_:Ar:H_2_ = 1.6 × 10^−7^:1.6 × 10^−5^:8.3 × 10^−6^;	P_atm_/873/**Annealing (5); Synthesis (15)**	~3	~100	5.1 × 10^12^	- Growth of double-/triple--walled VACNT - Two-point probe measurements present Ohmic contact with overall nanotubes-TiSiN support resistance of 0.7 ± 0.05 kΩ;	[90]
**Si wafer**	**8 nm Al on 100 nm TiN (Al or TiN targetS WITH 99.95% purity) by PVD DC magnetron sputtering** /Chamber pressure (0.7 Pa); Operating gas: Ar 25 + N (for TiN) (4.2 × 10^−7^ m^3^ s^−1^); PVD duration: 3 min Al+ 50 minTiN/3 nm Fe via DC magnetron sputtering	**photo-thermal**/C_2_H_2_ and H_2_	H_2_ (1.6 × 10^−6^); C_2_H_2_ (4.2 × 10^−7^)	(1.3 × 10^3^)/(~1023 catalyst surface; ~898-728 at bottom of Si substrate)/**Annealing (10);** **CNT Growth (15)**	~6 ± 3	473 ± 44	10^10^	- As grown VACNT network probe resistance: 10 Ω - Improved adhesion compared to Si wafer alone due to presence of Al_2_O_3_ top-layer	[60]
**p-type silicon wafers (100)**	**3 nm Co-Al (28%) on 50 nm TiN via sputtering**/-/-	**photo-thermal**/C_2_H_2_ and H_2_	- Annealing: H_2_ (1.2 × 10^−5^); - CNT Synthesis: H_2_ (1.2 × 10^−5^): C_2_H_2_ (8.3 × 10^−7^)	(8 × 10^3^)/(623)/**Annealing (60);** **CNT Growth (30)**	~8	~1.5	5 × 10^10^	- 60% of the tubes appear to be nanofibers	[71]
**Oxidized Si wafer**	**10 nm Al on 50 nm Mo via evaporation**/-/evaporation of 5 nm of Fe catalyst film	**pure-thermal**/C_2_H_4_	Only ration data: (H_2_:C_2_H_4_ = 1:3)	(-)/(993)/**(10)**	~30	~70	-	- CNT-covered supercapacitor - specific capacitance of 428 gF cm^−2^; - Excellent charge efficiency of 92% and robust cycling stability; - Device examined average power density = 0.28 mW cm^−2^	[34]

**Table 6:** Details of used substrate, DBL (deposited via ALD), and VACNT growth conditions and properties.

Substrate	DBL via ALD/Catalyst	Process of CVD/Carbon Source	Carrier or Carbon Source Gas: Flow Rate/Mixture (m^3^ s^−1^)	Pressure (Pa)/Temperature (K)/Reaction Duration (min)	CNT Average Diameter (nm)	CNT Length (µm)	Density of VACNT (Tubes cm^−2^)	DBL/VACNT/Composite Properties	Reference
silicon substrate	**tetrakis(dimethylamino)titanium (TDMAT) with H_2_O at 473 K for 20 nm DBL**/Fe catalyst deposition by electron-beam (EB) evaporation; Nominally 1 nm thick	**pure-thermal**/C_2_H_2_ and H_2_	- Annealing step: H_2_ (1.2 × 10^−5^) - CNT Synthesis: H_2_ (1.2 × 10^−5^):C_2_H_2_ (1.7 × 10^−6^)	-/773–1023/**Annealing (3); Synthesis (30)**	-	-	-	- Growth of four-walled VACNT	[93]
silicon substrate	**Diethyl zinc (DEZ) with H_2_O at 473 K for 20 nm DBL**/Fe catalyst deposition by electron-beam (EB) evaporation; Nominally 1 nm thick	**PECVD system radio frequency power of 75 W**/C_2_H_2_ and NH_3_	- CNT Synthesis: NH_3_ (2.7 × 10^−6^):C_2_H_2_ (6.7 × 10^−7^)	-/973–1173/**Synthesis (15)**	-	-	-	- Growth of multi-walled VACNT	[94]
Si coated with 200 nm of thermal SiO_2_	**Sequential exposures to TiCl and NH_3_ precursors at 473 K for 100 nm DBL with-/-out native oxide layer**/Fe catalyst deposition by thermal evaporation; Nominally 3–4–5 nm thick	**pure-thermal CVD**/undiluted C_2_H_2_	- Annealing step: H_2_ - CNT Synthesis: C_2_H_2_	**0.1–1**/873–973/**Annealing (5); Synthesis (15)**	4–7	-	-	- Growth of multi-walled VACNT. - Post-annealing density of Fe nanoparticles from 3 nm thick catalyst layer (2.6 ± 0.3) 10^11^ cm^−2^, with a lateral size distribution of 6 ± 2 nm	[86]

**Table 7:** Details of used carbonaceous substrates for direct growth of VACNT.

Substrate	Process of CVD/Carbon Source	Catalyst	Carrier or Carbon Source Gas: Flow Rate/Mixture (m^3^ s^−1^)	Pressure (Pa)/Temperature (K)/Reaction Duration (min)	CNT Average Diameter (nm)	CNT Length (µm)	Density of VACNT (Tubes cm^−2^)	DBL/VACNT/Composite Properties	Reference
100 nm amorphous carbon (a-C) on polished Si wafer with 300 nm SiO_2_	**pure-thermal**/C_2_H_4?_ Or C_2_H_2?_	- Fe catalyst deposition by thermal evaporation; Nominally 1 nm thick, evaporation rate of 0.1 Å s^−1^	- Annealing step: Ar (1.7 × 10^−5^) - CNT Synthesis: Ar (3.3 × 10^−6^):H_2_ (8.3 × 10^−6^):C_2_H_4_ (8.3 × 10^−6^)	**P_atm_**/773–1023 (60 K min^−1^)/**Annealing (10); Synthesis (10)**	Average 10–20	200 ± 17	~10^10^	- Growth of four-walled VACNT	[32]
Plasma oxidized HOPG	**alcohol catalytic**/C_2_H_5_OH vapor	- iron (Fe) or cobalt (Co) (2 nm) via arc plasma deposition	C_2_H_5_OH vapor (3.3 × 10^−6^)	**Co catalyst (10) or Fe catalyst (100)**/923–973/**10**	-	~1	6.3 × 10^10^	- Capacity: VACNT/HOPG 1.5 mF cm^−2^; Electric charge and discharge capacity: VACNT/HOPG 33.2 and 30.7 μQ cm^−2^	[5]
0.125-mm-thick graphite foil (Good fellow Corp., Coraopolis, PA, USA)	**plasma-enhanced**/C_2_H_2_	- Ni (60 nm) via radio frequency (RF)magnetron sputtering; - catalyst seeding process initiated by forming at 873 K for 30 min results in catalyst nanoparticles of ~140 nm	Only ration data: (C_2_H_2_:NH_3_ = 40:60)	**270**/973/**20**	~100	~3	-	- Flexibility test: No cracking or peeling off of CNT- Electrode potential: ΔEp (mV) = 66.07 at a scan rate of 10 mV s^−1^; Stable after 250 rolling cycles	[48]
Graphene paper (GP) Jiaxing Zhixianre Information Technology Co. Ltd., Jiaxing, China (thickness: ∼25 μm, density: 1.0 g cm^−3^, conductivity: 30,000 S cm^−1^, tensile strength: 40 MPa)	**injection CVD**/C_2_H_5_OH:C_2_H_8_N_2_ (volume ratio of 4:1)	- ferrocene (0.02 g mL^−1^) in C_2_H_5_OH:C_2_H_8_N_2_ (volume ratio of 4:1) - injected into the reactor at a rate of 10 mL h^−1^ through a thin tube by a syringe	Ar	-/885/-	-	~20	-	- CNT tube−tube distance of ~100 nm; - VACNT-GP-PDMS 4 layer composite with electrical conductivity stable at ~2800 S cm^−1^ after repeated vertical compression for 100 times under 50 N pressures, ultrahigh EMI SE of 106.7 dB, and tensile strength of 13.4 MPa and fracture strain 8.8% higher than the reference GP-PDMS 4-layer composite; - VACNT-GP-PDMS 4-layer composite tested as a high-performance Joule heater at low supplied voltages, rapid response time, and sufficient heating stability, with details given therein;	[49]

**Table 8:** Details of used carbonaceous fibrous substrates for direct growth of VACNT.

Substrate	Process of CVD/Carbon Source	Catalyst	Carrier or Carbon Source Gas: Flow Rate/Mixture (m^3^ s^−1^)	Pressure (Pa)/Temperature (K)/Reaction Duration (min)	CNT Average Diameter (nm)	CNT Length (µm)	Density of VACNT (Tubes cm^−2^)	DBL/VACNT/Composite Properties	Reference
(PAN)-based CF (Toho Tenax Co. Ltd., Chyoda City, Tokyo, Japan)	**pure-thermal**/C_6_H_6_	- 0.0001M solution (Fe(NO_3_)_3_ · 9H_2_O (SYSTEM)) in acetone by using ultrasonic-assisted impregnation (bath sonicator (200 W, 53 kHz)) - drying at ambient temperature for 24 h, calcination at 523 K for 120 min to decompose Fe(NO_3_)_3_ · 9H_2_O in air.	H_2_ (1.7 × 10^−6^)	**P_atm_**/973/-	≥20	≥5	-	-	[120]
CF paper TGPH090 (Toray Inc., Chyoda City, Tokyo, Japan)	**PECVD at a radio frequency power of 200 W**/CH_4_	- Fe(NO_3_)_3_ in ethanol - drying in a vacuum oven at 353 K	Ar (3.3 × 10^−6^):H_2_ (6.7 × 10^−7^):CH_4_ (1.3 × 10^−6^)	**P_atm_**/973/**30**	Inner (5), Outer (15)	6–10	-	- Used as an electrode for electro-oxidation of methanol; potential of the methanol oxidation peak—0.6 V; current density of the methanol oxidation peak 660 mA/(cm^2^ mg)	[123]
UTS50S CF, TeXtreme	**pure-thermal**/C_2_H_2_	- Fe(NO_3_)_3_ · 9H_2_O in iso-propanol (0.2 mol/L) - applied 5 times with an interval of 3.5 min, by pipette, to the CF surface (fibers curved under an angle), and the excess catalyst drained under gravity. - dried at room temperature for 15 h and then at 368 K for 3 h	- Ar (1.7 × 10^−5^)—ramp period; dwell 5 min; - He (1.7 × 10^−5^)—2 min, then mixture (He:C_2_H_2_:H_2_ 1.6 × 10^−5^: 3.3 × 10^−7^: 3.3 × 10^−7^); - End: dwell He (1.7 × 10^−5^)—5 min; - Cooling: Ar (1.7 × 10^−5^)	-/973 (45 K min^−1^)/**13**	-	~2–3	-	- Transverse conductance: UTS50S/CNT (1µM height) = 0.11 S cm^−1^ (through-thickness) UTS50S/CNT (2µM height) = 0.22 S cm^−1^ (through-thickness)	[125]

**Table 9:** Details of used CF substrates for growth of VACNT with the use of CVD/PVD deposited DBL.

Substrate	DBL/Thickness (nm)/**Deposition technique**/Precursor/Temp. (K), Duration (min), Pressure (Pa)	**Catalyst**	**Process** **of CVD**/Carbon Source	Carrier or Carbon Source Gas: Flow Rate/Mixture (m^3^ s^−1^)	**Pressure (Pa)**/Temperature (K)/**Reaction Duration (min)**	CNT Average Diameter (nm)	CNT Length (µm)	Density of VACNT (Tubes cm^−2^)	DBL/VACNT/Composite Properties	Reference
1K Carbon cloth T300 CF	pyrolithic carbon/(~200)/**CVD**/C_2_H_5_OH vapour with CH_4_/(1423), (120), (100–1 × 10^5^)	FeSO_4_·7H_2_O solution (20 kg m^−3^); Pulsed electro-deposition for 5 min	**pure-thermal**/CH_4_	CH_4_ (4.2 × 10^−6^): Ar (4.2 × 10^−5^)	-/(1423)/**(120)**	Inner (70), Outer (150)	-	-	- CNT Improved adhesion on DBL	[45]
Anodized Aluminum Oxide Template	Graphitic layer/-/**CVD**/C_2_H_2_ in Ar (5:30)/(923), (-), (P_atm_)	1.5 nm Fe layer via E-beam evaporation	**pure-thermal**/CH_4_ and H_2_O vapors	-	**P_atm_**/923/-	-	5–10	-	- VACNT−carbon nanocups based supercapacitor exhibited a specific capacitance of 0.6 mF/cm^2^; - Cyclic stability up to 10,000 charge/discharge cycles; - Resistance of 23 Ω between the VACNT−carbon nanocups electrode and a gold current collector.	[92]
Carbon felts (Yixing Tianniao Co., Yixing, Jiangsu, China)	pyrolithic carbon/dozens nm/**CVI**/CH_4_ diluted in N_2_/(1343), (-), (P_atm_)	1.5 wt.% Ni(NO_3_)_2_·6H_2_O in acetone by impregnation for 12 h	**pure-thermal**/CH_4_	CH_4_ (4.5 × 10^−6^): Ar (3.3 × 10^−5^)	-/(1323)/**(120)**	-	8–20 on periphery; 1–5 in interior of felt	-	- Compared to lone C/C composite: ->+275% out-of-plane compression strength, ->+138% in-plane compression strength; ->+206% interlaminar shear strength; ->+125% out-of-plane compression modulus; ->+65% out-of-plane compression modulus;	[130]
Carbon paper	Al—Ti/(2 nm)-(5 nm)/**electron beam PVD**/-/(-), (-), (-)	Fe (1 nm) electron beam PVD	**hot filament** assisted/C_2_H_2_	Only ration data: (H_2_:C_2_H_2_ = 157:10)	**120**/(773, 50 K min^−1^; cooling under H_2_)/**(30)**	7.5–8	10–20	-	- Voltage 0.75 V; current densities (1.1 A cm^−2^); details are given for the sample size and single-cell test	[47]
CF Pyrofil TR30s (Grafil Inc., Sacramento, CA, USA)	Al/(35 nm)/**PVD DC magnetron sputtering**/-/(-), (-), (-)	Fe (6 nm) via DC magnetron sputtering	**PT-CVD** system/C_2_H_2_	H_2_ (1.7 × 10^−6^):C_2_H_2_ (3.3 × 10^−7^)	**1333**/(Catalyst activation step (1023); Synthesis VACNT (1073)/**(10)**	12.5	10–300	~2 × 10^10^	- Electrical resistance of VACNT/CF reinforced polymers—CFRP (in-plane ~ 55 Ω; through-thickness ~ 5555 Ω; through-volume ~ 62 Ω)	[4]

## Data Availability

The data in this study are available on reasonable request from the corresponding author.
